# Glycosylated and Succinylated Macrocyclic Lactones with Amyloid-β-Aggregation-Regulating Activity from a Marine *Bacillus* sp.

**DOI:** 10.3390/md21020067

**Published:** 2023-01-19

**Authors:** Jinsheng Cui, Suhyun Ye, Daniel Shin, Illhwan Cho, Hye Yun Kim, Yun Kwon, Keunwan Park, Sang-Jip Nam, YoungSoo Kim, Dong-Chan Oh

**Affiliations:** 1 Natural Products Research Institute, College of Pharmacy, Seoul National University, Seoul 08826, Republic of Korea; 2 Department of Pharmacy and Yonsei, Institute of Pharmaceutical Sciences, College of Pharmacy, Yonsei University, Incheon 21983, Republic of Korea; 3 Research Institute of Pharmaceutical Science, College of Pharmacy, Kyungpook National University, Daegu 41566, Republic of Korea; 4 Natural Product Informatics Research Center, Korea Institute of Science and Technology, Seoul 25451, Republic of Korea; 5 Department of Chemistry and Nanoscience, Ewha Womans University, Seoul 03760, Republic of Korea

**Keywords:** glycosylation, succinylation, macrocyclic lactone, absolute configuration, *Bacillus* sp., amyloid-β

## Abstract

Two new glycosylated and succinylated macrocyclic lactones, succinyl glyco-oxydifficidin (**1**) and succinyl macrolactin O (**2**), were isolated from a *Bacillus* strain collected from an intertidal mudflat on Anmyeon Island in Korea. The planar structures of **1** and **2** were proposed using mass spectrometric analysis and NMR spectroscopic data. The absolute configurations of **1** and **2** were determined by optical rotation, *J*-based configuration analysis, chemical derivatizations, including the modified Mosher’s method, and quantum-mechanics-based calculation. Biological evaluation of **1** and **2** revealed that succinyl glyco-oxydifficidin (**1**) inhibited/dissociated amyloid β (Aβ) aggregation, whereas succinyl macrolactin O (**2**) inhibited Aβ aggregation, indicating their therapeutic potential for disassembling and removing Aβ aggregation.

## 1. Introduction

Glycosylation is an important biological process in enhancing the structural and biological diversity of metabolites [1,2]. In many cases, the biological function of natural products is altered after glycosylation; glycosylation is thus a frequently adopted route to the functional modification of molecules in nature [3]. For example, a study on the effects of glycosylation on the biological activity of the microbial immunosuppressive drug rapamycin revealed that glycosylation improved water solubility and reduced cytotoxicity depending on its positions [4]. A chemical investigation of *Bacillus* sp. reported glycosylated macrolactins, which displayed inhibitory activity against *Staphylococcus aureus* peptide deformylase along with antibacterial activity differing from that of previously reported aglycone macrolactins, indicating that the discovery of glycosylated natural products would lead to the diversification of their biological functions [5].

Marine bacteria are also fruitful sources of structurally and biologically diverse natural product discovery [6]. Since the 21st century, more and more drug candidates were discovered from Gram-positive bacteria such as *Streptomyces* sp., *Bacillus* sp. and so on [7]. Our chemical studies on marine bacteria discovered pulvomycins B–D, new macrolides incorporating sugar, and pulvomycin D showed potent cytotoxic effects against cancer cell lines [8]. Suncheonosides A–D, hexasubstituted benzothioate glycosides, were reported to be promotors of adiponectin production from a marine sediment-derived *Streptomyces* sp. [9]. Our recent genomic and spectroscopic analysis of a marine sand-beach-derived *Streptomyces* strain resulted in the discovery of a jejucarboside bearing an unusual amino sugar [10]. *Bacillus* in marine habitats has also been a chemically prolific bacterial clade since new antiviral and cytotoxic macrolides, macrolactins, were reported from deep-sea *Bacillus* strain [11]. Continuous chemical investigation led to discover antimicrobial glycopeptides, ieodoglucomides, from marine *B. licheniformis* [12], algicidal thiazole-bearing compounds, bacillamides, from marine *Bacillus* sp. [13], disulfide-bearing antimicrobial lipoamides from marine *B. pumilus* [14], and antifungal basiliskamide from tropical marine *B. laterosporus* [15], respectively.

In our continuous research, we have chemically profiled a marine strain of *Bacillus* sp. AMD05, which was isolated from an intertidal mudflat in Anmyeondo, Republic of Korea, and discovered two new glycosylated and succinylated macrolides, named succinyl glyco-oxydifficidin (**1)** and succinyl macrolactin O (**2**). Combinational analysis of mass, NMR, and UV spectroscopic data enabled us to elucidate the structures of **1** and **2**. We further applied chiral derivatization, the modified Mosher’s method, and quantum mechanics-based DP4 probability calculation to determine the absolute configuration of succinyl glyco-oxydifficidin (**1**), which had previously been unknown. In this study, we report the structure elucidation of **1** and **2** and their biological evaluation in Alzheimer’s-disease-related assays.

## 2. Results and Discussion

### 2.1. Structure Elucidation

**Figure 1:**
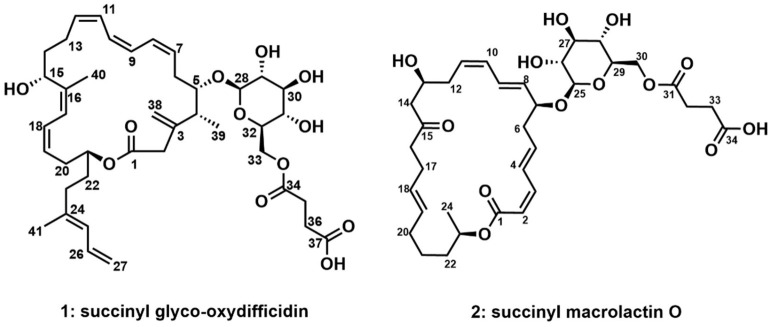
Structures of succinyl glyco-oxydifficidin (**1**) and succinyl macrolactin O (**2**).

Succinyl glyco-oxydifficidin (**1**) was isolated as a yellow powder. The molecular formula of **1** was deduced as C_41_H_58_O_12_ based on its HRESIMS data. Its molecular formula revealed 13 degrees of unsaturation. Its UV spectrum (λ_max_: 234, 274, and 284 nm) indicated the existence of a diene and a triene chromophore in **1** [16]. All the ^1^H–^13^C one-bond correlations in succinyl glyco-oxydifficidin (**1**) were assigned by combined analysis of ^1^H, ^13^C, and HSQC NMR spectra. Eleven sp^2^ methine protons (*δ*_H_ 6.584–4.969), four sp^2^ methylene protons, one proton (*δ*_H_ 4.285) bound to a dioxygenated carbon (*δ*_C_ 105.91), seven carbinol methine protons (*δ*_H_ 4.802, 4.680, 3.693, 3.420, 3.315, 3.267, and 3.188), and two oxygenated methylene protons (*δ*_H_ 4.405 and 4.220) were identified. One methine proton (*δ*_H_ 2.369), eighteen protons belonging to nine methylene groups (*δ*_H_ 3.444, 3.059, 2.732, 2.601, 2.596 (2H), 2.546 (2H), 2.512, 2.403, 2.311, 2.102 (2H), 1.945, 1.817, 1.803, 1.739, 1.703), and three methyl groups (*δ*_H_ 1.802 (3H), 1.779 (3H), 0.979 (3H)) were also assigned in the aliphatic region. However, four hydroxy protons and one carboxyl acid proton were not observed in CD_3_OD. The ^13^C NMR data revealed three ester or carboxylic acid group carbons (*δ*_C_ 177.20, 174.37, and 174.03), sixteen double-bond carbons (*δ*_C_ 148.67–113.99), one dioxygenated carbon (*δ*_C_ 105.91), eight mono-oxygenated carbons, including seven oxygenated methine carbons (*δ*_C_ 86.94–68.14), and one oxygenated methylene carbon (*δ*_C_ 64.93), one aliphatic methine carbon (*δ*_C_ 41.19), nine aliphatic methylene carbons (*δ*_C_ 47.03, 36.75, 34.10, 33.45, 32.29, 32.03, 30.90, 30.64, and 29.18), and three methyl carbons (*δ*_C_ 17.76, 17.40, and 16.62) in the structure of **1** (Figure 1).

**Figure 2:**
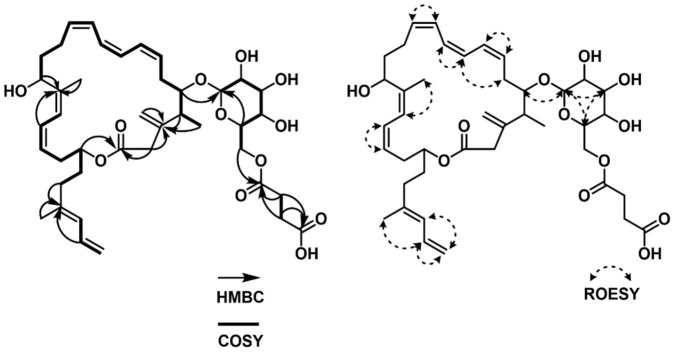
COSY, HMBC and ROESY correlations of succinyl glyco-oxydifficidin (**1**).

Via analyzing the COSY NMR data of **1**, several spin systems were identified. The spin system from C-4 (*δ*_C_ 41.19) to C-15 (*δ*_C_ 68.14), including the C-39 methyl group (*δ*_C_ 17.40) at C-4, was straightforwardly assembled by a series of COSY correlations from H_3_-39 to H-15. The second spin system of C-17 (*δ*_C_ 123.89) to C-23 (*δ*_C_ 36.75) was revealed by consecutive ^1^H-^1^H couplings from H-17 (*δ*_H_ 6.232) to H_2_-23 (*δ*_H_ 2.102 (2H)) through H-18 (*δ*_H_ 6.208), H-19 (*δ*_H_ 5.257), H_2_-20 (*δ*_H_ 2.601 and 2.403), H-21 (*δ*_H_ 4.802), and H_2_-22 (*δ*_H_ 1.817 and 1.703). A three-carbon connection in the tail of the carbon backbone, C-25 (*δ*_C_ 127.37)–C-26 (*δ*_C_ 134.48)–C-27 (*δ*_C_ 115.53), was also elucidated using the COSY correlations of H-25 (*δ*_H_ 5.861), H-26 (*δ*_H_ 6.584), and H_2_-27 (*δ*_H_ 5.071 and 4.969). Another spin system of the hexose was connected based on the ^1^H-^1^H couplings from H-28 (*δ*_H_ 4.285) to H_2_-33 (*δ*_H_ 4.405 and 4.260) via H-29 (*δ*_H_ 3.188), H-30 (*δ*_H_ 3.315), H-31 (*δ*_H_ 3.267), and H-32 (*δ*_H_ 3.420). C-35 (*δ*_C_ 30.90) and C-36 (*δ*_C_ 30.64) were linked by the H_2_-35 (*δ*_H_ 2.546 (2H))/H_2_-36 (*δ*_H_ 2.596 (2H)) COSY correlation (Figure 2).

These partial structures were assembled together by interpretation of HMBC NMR data (Figure 2). The ^2^*J*_CH_ and ^3^*J*_CH_ HMBC correlations from H-4 (*δ*_H_ 2.369), H_3_-39 (*δ*_H_ 0.979), and H_2_-2 (*δ*_H_ 3.059 and 3.444) to C-3 (*δ*_C_ 148.67) constructed the C-2–C-3–C-4 linkage, while the HMBC correlation of olefinic protons, H_2_-38a (*δ*_H_ 5.026) and H_2_-38b (*δ*_H_ 5.046), to C-3 (*δ*_C_ 148.67) connected the olefinic methylene to C-3. HMBC correlations from H-15 (*δ*_H_ 4.680) and H-17 (*δ*_H_ 6.232) to C-16 (*δ*_C_ 140.85) constructed the C-15–C-16–C-17 connectivity, leading to the two spin systems from C-2 to C-23 merging together. A methyl group was attached to the olefinic C-16 by an HMBC correlation from H_3_-40 (*δ*_H_ 1.802) to C-16 (*δ*_C_ 140.83). A four-olefinic-carbon tail was connected to C-23 by HMBC correlations from H-23 (*δ*_H_ 2.102) and H-26 (*δ*_H_ 6.582) to C-24 (*δ*_C_ 139.27). The HMBC correlation of the methyl protons H_3_-41 (*δ*_H_ 1.779) to C-24 (*δ*_C_ 139.27) revealed the connection between C-41 and C-24. The macrocyclic lactone skeleton was constructed by the HMBC correlations of H_2_-2 (*δ*_H_ 3.059 and 3.444) and H-21 (*δ*_H_ 4.802) to C-1 (*δ*_C_ 174.03). Furthermore, the glycosyl group was assigned to the macrocycle by the H-5 (*δ*_H_ 3.693)/C-28 (*δ*_C_ 105.91) HMBC correlation. The succinate moiety, which was revealed by the HMBC correlations of H_2_-35 (*δ*_H_ 2.546 (2H)) and H_2_-36 (*δ*_H_ 2.596 (2H)) to C-34 (*δ*_C_ 174.37) and C-37 (*δ*_C_ 177.20), was linked to the sugar moiety by the HMBC correlation of H_2_-33 (*δ*_H_ 4.405 and 4.220) to C-34 (*δ*_C_ 174.37). Therefore, the planar structure of succinyl glyco-oxydifficidin (**1**) was determined to be a new succinyl glycosyl macrolactone, as shown in Figure 1.

The double-bond geometry configurations of **1** were determined by ^1^H-^1^H coupling constants and ROESY correlations. The ^3^*J*_H7H8_ value (11.0 Hz) determined the 7*Z* configuration, which was also supported by the H-7 (*δ*_H_ 5.639)/H-8 (*δ*_H_ 6.440) and H-6b (*δ*_H_ 2.732)/H-9 (*δ*_H_ 6.162) ROESY correlations. H-9 (*δ*_H_ 6.162)/H-10 (*δ*_H_ 5.998) and H-11 (*δ*_H_ 6.439)/ H-12 (*δ*_H_ 5.707) ROESY correlations assigned 9*Z* and 11*Z* configurations. 16*Z* and 18*Z* geometries were established by H_3_-40 (*δ*_H_ 1.802 (3H))/ H-17 (*δ*_H_ 6.232) and H-18 (*δ*_H_ 6.208)/ H-19 (*δ*_H_ 5.257) ROESY correlations (Figure 2). Lastly, the H_3_-41/H-26 ROESY correlation determined the 24*E* configuration.

**Figure 3:**
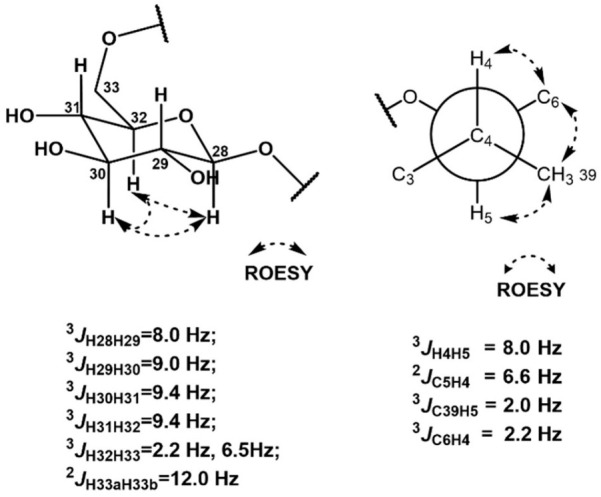
*J* coupling constant values and ROESY correlations of the sugar and C4–C5 moieties.

The relative configuration of the sugar moiety was also assigned by ^1^H-^1^H coupling constants and ROESY correlations [16]. H-28 (*δ*_H_ 4.285) and H-29 (*δ*_H_ 3.188) were located at axial positions by the large coupling constant value of ^3^*J*_H28H29_ (8.0 Hz). The coupling constant of 9.0 Hz between H-29 (*δ*_H_ 3.188) and H-30 (*δ*_H_ 3.315) also indicated their axial–axial relationship. The large value (9.4 Hz) of both ^3^*J*_H30H31_ and ^3^*J*_H31H32_ revealed the assignment of H-30 (*δ*_H_ 3.315), H-31 (*δ*_H_ 3.267), and H-32 (*δ*_H_ 3.420) at axial positions of the sugar. Furthermore, the ROESY correlations of H-28 (*δ*_H_ 4.285)/H-30 (*δ*_H_ 3.315), H-30 (*δ*_H_ 3.315)/H-32 (*δ*_H_ 3.420), and H-28 (*δ*_H_ 4.285)/H-32 (*δ*_H_ 3.420) assigned these three protons in the same plain, and thus the sugar was determined to be β-glucose. In addition, ^3^*J*_H32H33a_ (2.2 Hz), ^3^*J*_H32H33b_ (6.5 Hz), and ^3^*J*_H33aH33b_ (12.0 Hz) supported β-glucose (Figure 3).

The relative configuration between C-4 and C-5 was revealed by *J*-based configuration analysis [17]. Long-range ^13^C−^1^H coupling constants were measured by hetero-half-filtered TOCSY (HETLOC) NMR experiments [18]. The large coupling constant of ^3^*J*_H4H5_ (8.0 Hz) indicated an *anti*-relationship between H-4 (*δ*_H_ 2.369) and H-5 (*δ*_H_ 3.693). The small value of ^3^*J*_C39H5_ (2.0 Hz) assigned a gauche position of the C-39 (*δ*_C_ 17.40) methyl group to H-5 (*δ*_H_ 3.693). Furthermore, the small coupling constant (2.2 Hz) between C-6 (*δ*_C_ 32.03) and H-4 (*δ*_H_ 2.369) established their gauche relationship. In addition, the coupling constant of ^2^*J*_C5H4_ (6.6 Hz) and ROESY correlations of H-4 (*δ*_H_ 2.369)/H-6a (*δ*_H_ 2.512), H_3_-39 (*δ*_H_ 0.979)/H-6b (*δ*_H_ 2.732), and H_3_-39 (*δ*_H_ 0.979)/H-5 (*δ*_H_ 3.693) revealed the 4R*,5S* relative configuration (Figure 3).

Once the assignment of β-glucose was established, chemical derivatization was conducted for the absolute configuration of the glucose [19]. We used acid hydrolysis to break the connection between the corresponding aglycone with glucopyranose. β-glucopyranose was assigned as β-d-glucopyranose by chiral derivatization with l-cysteine methyl ester hydrochloride and σ-tolyl isothiocyanate and subsequent LC/MS analysis (Figure S16).

**Figure 4:**
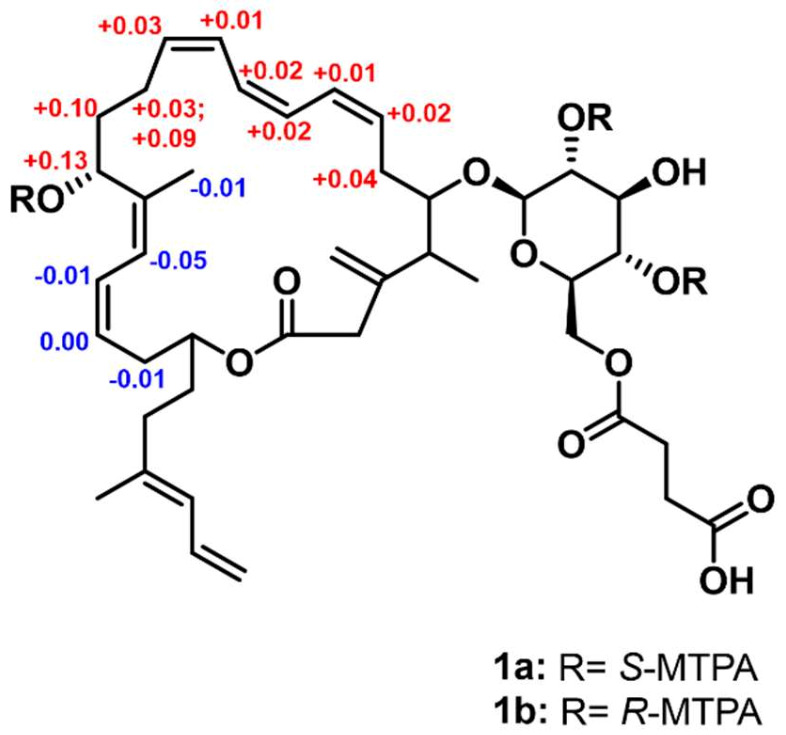
Δ*δ_S–R_* values (ppm) of *S*- and *R*-MTPA esters of **1**.

The absolute configuration of the stereogenic center at C-15, which bears a secondary alcohol, was determined using the modified Mosher’s method [20]. The hydroxy groups at C-15 were esterified with *R*- and *S*-α-methoxy-α-(trifluoromethyl) phenylacetyl chloride (MTPA-Cl) to the tri-*S*- and *R*-MTPA esters (**1a** and **1b**). The calculated ^1^H-NMR spectroscopic Δ*δ_S__−__R_* values established the absolute configuration as 15*R* (Figure 4 and Figures S8–S11).

When it is limited to deduce the configurations of organic compounds by application of NMR spectroscopic analysis and chemical derivatization methods, quantum mechanics-based computational approaches, including the advanced probabilistic methods including CP3 and DP4 calculations, can be utilized [21]. DP4 calculations were then applied to establish the absolute configuration for the remaining chiral centers of C-4, C-5, and C-21 [22]. Four possible diastereomers of **1** simplified without the succinic acid (**1c** (4*R*, 5*S*, and 21*S*), **1d** (4*R*, 5*S*, and 21*R*), **1e** (4*S*, 5*R*, and 21*S*), and **1f** (4*S*, 5*R*, and 21*R*)) were constructed using Avogadro 3D modeling program. Then, the ^1^H and ^13^C NMR chemical shifts of the four conformers whose relative potential energy was below 10 kJ/mol were calculated and averaged with their Boltzmann populations. The computational NMR shielding of **1c**, **1d**, **1e,** and **1f** was calculated using TmoleX 4.2.1. **1c** (4*R*, 5*S*, and 21*S*) achieved 100.0% probability based on statistical comparison of the calculated and experimental chemical shifts using DP4 calculation (https://www-jmg.ch.cam.ac.uk/tools/nmr/DP4/, accessed on 13 September 2020). (Tables S1 and S2, Figure S17), completing the structure elucidation of succinyl glyco-oxydifficidin (**1**).

Succinyl macrolactin O (**2**) was isolated as a yellow powder. The molecular formula of **2** was deduced as C_34_H_48_O_13_ based on its HRESIMS data. Its molecular formula revealed 11 degrees of unsaturation. The UV spectrum (λ_max_: 236 and 260 nm) of **2** indicated at least two chromophores in the structure. By careful analysis of ^1^H, ^13^C, and HSQC NMR spectra, all the ^1^H–^13^C one-bond correlations in **2** were identified. Ten olefinic protons (*δ*_H_ 7.229–5.411), eight oxygen-bound methine protons (*δ*_H_ 5.001, 4.370, 4.309, 4.118, 3.405, 3.328, 3.300, 3.239), and two oxygenated methylene protons (*δ*_H_ 4.423 and 4.250) were identified. Twenty protons belonging to ten methylene groups (*δ*_H_ 2.654 (2H), 2.605 (2H), 2.580, 2.555 (2H), 2.463 (2H), 2.461 (2H), 2.373, 2.197 (2H), 2.051, 1.959, 1.630, 1.531, 1.428, 1.393) and one methyl group (*δ*_H_ 1.232 (3H)) were also identified using the ^1^H NMR and HSQC data. The ^13^C NMR data revealed one ketone carbon (*δ*_C_ 211.82), three ester or carboxyl acid groups (*δ*_C_ 176.15, 174.21, and 167.97), ten olefinic carbons (*δ*_C_ 145.37–117.83), nine oxygenated carbons, including eight oxygenated methine carbons (*δ*_C_ 101.37–68.66), and one oxygenated methylene carbon (*δ*_C_ 64.94), ten methylene carbons (*δ*_C_ 49.42–26.19), and one methyl carbon (*δ*_C_ 20.26) in the structure of **2**.

**Figure 5:**
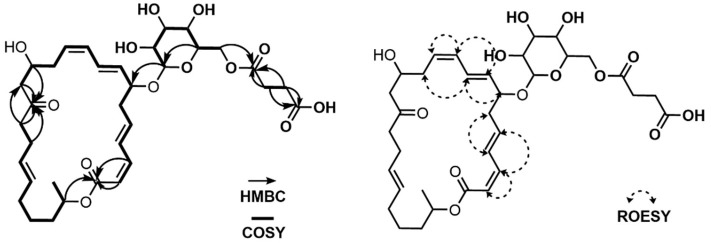
COSY, HMBC and ROESY correlations of succinyl macrolactin O (**2**).

The spin system from C-2 (*δ*_C_ 117.83) to C-14 (*δ*_C_ 49.42) could be straightforwardly connected by a series of COSY correlations from H-2 to H_2_-14 through H-3 (*δ*_H_ 6.628), H-4 (*δ*_H_ 7.229), H-5 (*δ*_H_ 6.214), H_2_-6 (*δ*_H_ 2.560, 2.456), H-7 (*δ*_H_ 4.370), H-8 (*δ*_H_ 5.636), H-9 (*δ*_H_ 6.581), H-10 (*δ*_H_ 6.146), H-11 (*δ*_H_ 5.545), H_2_-12 (*δ*_H_ 2.463, 2.373), and H-13 (*δ*_H_ 4.118). The second spin system of C-16–C-24 was revealed by the consecutive COSY correlations from H-16 (*δ*_H_ 2.461) to H_2_-24 (*δ*_H_ 1.232) via H-17 (*δ*_H_ 2.197), H-18 (*δ*_H_ 5.411), H-19 (*δ*_H_ 5.411), H_2_-20 (*δ*_H_ 2.052, 1.959), H_2_-21 (*δ*_H_ 1.428, 1.393), H_2_-22 (*δ*_H_ 1.630, 1.531), H-23 (*δ*_H_ 5.001), and H_3_-24 (*δ*_H_ 1.232). The third spin system, C-25–C-30, was also constructed based on COSY correlations from H-25 (*δ*_H_ 4.309) to H_2_-30 (*δ*_H_ 4.423, 4.250). C-32 and C-33 were linked as the last spin system by their COSY correlation H_2_-32 (*δ*_H_ 2.605 (2H))/H_2_-33 (*δ*_H_ 2.654 (2H)) (Figure 5). These substructures were connected by key HMBC correlations; the H_2_-14 (*δ*_H_ 2.580, 2.555)/C-15 (*δ*_C_ 211.82) and H-16 (*δ*_H_ 2.461)/C-15 (*δ*_C_ 211.82) correlations connected the piece C-14–C-15–C-16, therefore constructing the C-2–C-24 chain skeleton. H-2 (*δ*_H_ 5.544)/C-1 (*δ*_C_ 167.97) and H-3 (*δ*_H_ 6.628)/C-1 (*δ*_H_ 167.97) heteronuclear correlations revealed the C-1–C-2 linkage, while an ester bond was established by H-23 (*δ*_H_ 5.001)/C-1 (*δ*_H_ 167.97) coupling, completing the 24-membered macrocyclic skeleton. The glucoside, which was confirmed by H-29 (*δ*_H_ 3.402)/C-25 (*δ*_C_ 101.37) correlation, was connected to C-7 based on the HMBC correlation of H-25 (*δ*_H_ 4.309) to C-7 (*δ*_H_ 78.73). A succinate moiety was revealed by H_2_-32 (*δ*_H_ 2.605 (2H))/C-31 (*δ*_C_ 174.21), H_2_-33 (*δ*_H_ 2.654 (2H))/C-31 (*δ*_C_ 174.21), H_2_-32 (*δ*_H_ 2.605(2H))/C-34 (*δ*_C_ 176.15), and H_2_-33 (*δ*_H_ 2.654 (2H))/C-34 (*δ*_C_ 176.15) correlations. This succinate moiety was attached to the glucoside by H_2_-30 (*δ*_H_ 4.423, 4.250)/C-31 (*δ*_C_ 174.21) HMBC correlation (Figure 5). Therefore, the planar structure of **2** was proposed, as shown in Figure 1.

Analogously to **1**, the sugar moiety in **2** was assigned as β-glucose by ^1^H-^1^H coupling constants and ROESY correlations. Its absolute configuration was revealed as β- d-glucopyranose by the chiral derivatization and LC/MS analysis (Figure S28). As many macrolactin derivatives were discovered and their absolute configurations are conserved in the family, we compared the [α]_D_ values of succinyl macrolactin O (**2**) ([α]_D_ = −36.8 (c 0.1, MeOH)) with those of the most closely related compound, macrolactin O (−56.8 (c 0.1, MeOH)), and proposed that succinyl macrolactin O (**2**) shared the same absolute configuration with macrolactin O [5].

### 2.2. Biological Activity

**Figure 6:**
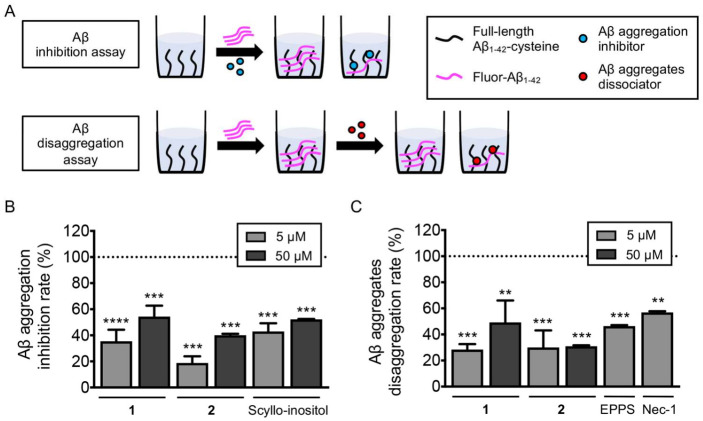
(**A**) Scheme of Aβ inhibition and dissociation assay. Investigation of the anti-amyloidogenic ability of succinyl glyco-oxydifficidin (**1**) and succinyl macrolactin O (**2**). (**B**,**C**) Aβ aggregation inhibition and Aβ aggregate dissociation assays were performed with the treatment of succinyl glyco-oxydifficidin and succinyl macrolactin O. The error bars represent the SEM and the statistical analyses were performed by one-way ANOVA analysis followed by Bonferroni’s post-hoc comparison to wells without fluorescent Aβ_1-42_ (dotted line). ** *p* < 0.01, *** *p* < 0.001, **** *p* < 0.0001. Fluor-Aβ_1-42_, fluorescent Aβ_1-42_; Nec-1, nectrostatin-1.

According to the previous studies, the antimicrobial activities of difficidin [23] and macrolactin [11] families were reported. However, we could not find any activity of **1** and **2** in our antimicrobial assays (Tables S4–S6). Therefore, we searched for unreported activity of the difficidin and macrolactin families and targeted amyloid-β-aggregation-regulating activity for these two compounds. Amyloid-β (Aβ) aggregates in the brain of patients with Alzheimer’s disease (AD) are considered the pathological and biological hallmarks of this neurodegenerative disorder [24]. Thus, drug candidates have been discovered to inhibit and reverse the Aβ aggregation process [25]. To investigate whether our compounds, succinyl glyco-oxydifficidin (**1**) and succinyl macrolactin O (**2**), inhibit and/or reverse Aβ aggregation, we performed two different types of assays, inhibition and dissociation, utilizing a high throughput screening platform we recently developed [26]. For both assays, we immobilized Aβ_1-42_ with an additional C-terminal cysteine on the maleimide-coated 96-well plate and added fluorescent Aβ_1-42_ to induce on-plate oligomer and fibril formation of Aβ (Figure 6A). First, we assessed **1** and **2**, in Aβ aggregation inhibition assay (Figure 6A). Each compound, at concentrations of 5 and 50 µM, was added to the Aβ_1-42_-immobilized plate with the fluorescent Aβ_1-42_ (10 µM) and incubated for 24 h at room temperature (RT) to observe in situ aggregation inhibition. We used a previously reported Aβ aggregation inhibitor, (1r,2r,3r,4r,5r,6r)-cyclohexane-1,2,3,4,5,6-hexol (scyllo-inositol), as a positive control [27]. The plate was washed after the incubation step, and we measured the levels of the remaining fluorescent Aβ_1-42_. Data were then normalized to the signal of fluorescent Aβ_1-42_-free wells as 100% inhibition of Aβ aggregation, and the inhibition rate (%) was analyzed as previously reported [26]. As a result, both compounds inhibited Aβ aggregation significantly; **1** by 35.54% (at 5 µM) and 54.35% (at 50 µM), and **2** by 18.88% (at 5 µM) and 40.08% (at 50 µM), when the control compound was inhibited by 43.00% (at 5 µM) and 52.22% (at 50 µM) (Figure 6B).

We further examined the compounds ability to dissociate pre-formed Aβ aggregates. In this assay, compounds were added to the plate after on-plate oligomer and fibril formation was induced [26]. Briefly, fluorescent Aβ_1-42_ was added to the Aβ_1-42_-immobilized plate and incubated for 8 h at 37 °C. Then, each compound in two concentrations, 5 and 50 µM, was treated to the plate and incubated for additional 24 h at RT (Figure 6A). Previously reported Aβ aggregate dissociators, 4-(2-hydroxyethyl)-1-piperazinepropanesulphonic acid (EPPS) [28] and 5-(1*H*-indol-3-ylmethyl)-3-methyl-2-thioxo-4-imidazolidinone (Necrostatin-1, Nec-1) [29], were used as positive controls. The plate was washed after the incubation step, and we measured the levels of the remaining fluorescent Aβ_1-42_. Fluorescent signal of wells without fluorescent Aβ_1-42_ treatment was regarded as 100% disassociation rate [26]. As a result, both compounds reversed Aβ aggregation significantly; **1** by 28.46% (at 5 µM) and 49.27% (at 50 µM), and **2** by 30.04% (at 5 µM) and 31.02% (at 50 µM), when the control compounds dissociated pre-formed aggregates by 46.42% (EPPS at 5 µM) and 59.94% (Nec-1 at 5 µM) (Figure 6C).

**Figure 7:**
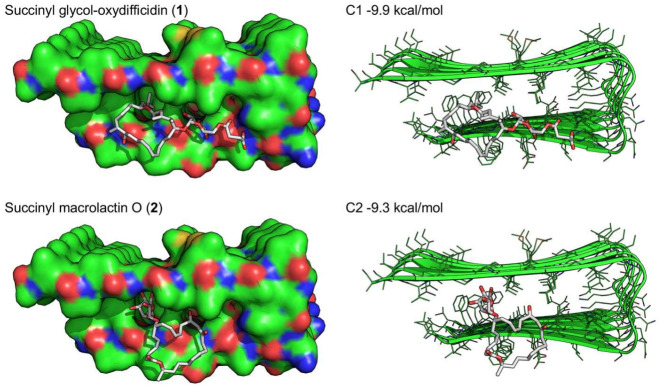
Docking simulations of succinyl glyco-oxydifficidin (**1**) and succinyl macrolactin O (**2**) docking into the U-shaped oligomeric Aβ (1-42) structure (PDB ID: 2BEG).

Next, we docked compounds **1** and **2** to the U-shaped oligomeric Aβ_1-42_ structure (PDB ID: 2BEG) to predict the potential binding interactions between them (Figure 7). The docking score of **1** (−9.9 kcal/mol) was slightly better than that of **2** (−9.3 kcal/mol). The docking models suggested that the branched carbon chain of **1**, which is not present in **2**, contributes to forming extensive hydrophobic contacts with the core of Aβ aggregate. Also, the additional contacts of **1** to the edge strand of Aβ aggregate seem to be a primary factor for inhibiting or dissociating Aβ aggregation. In contrast, **2** showed similar but relatively unfavorable interactions with the Aβ hydrophobic core through the sugar moiety (Figure 7).

Overall, we observed that succinyl glyco-oxydifficidin (**1**) dose-dependently inhibited/dissociated Aβ aggregation, whereas succinyl macrolactin O (**2**) dose-dependently inhibited Aβ aggregation. In these assays, we assumed that our scaffold had the therapeutic potential to disassemble and remove Aβ aggregation.

## 3. Materials and Methods

### 3.1. General Experimental Procedures

Optical rotations were measured by a Jasco P-2000 polarimeter with a 1.0 cm cell (Tokyo, Japan). UV and CD spectra were recorded using an applied photophysics Chirascan plus spectrometer (Leatherhead, UK). IR spectra were acquired with a JASCO FT/IR-4200 spectrometer (Tokyo, Japan). ^1^H, ^13^C, and 2D NMR spectra were obtained on Bruker Avance 800 MHz NMR spectrometers (Billerica, MA, USA), all the signals being referenced to ^13^C (49.045 ppm) and ^1^H (3.306 ppm) signals of CD_3_OD [30]. Electrospray ionization source (ESI) low-resolution LC/MS data were collected on an Agilent Technologies 6130 quadrupole mass spectrometer (Santa Clara, CA, USA) coupled with an Agilent Technologies 1200 series HPLC using a reversed-phase C_18_(2) column (Phenomenex Luna, 5 μm, 4.6 × 100 mm, Torrance, CA, USA). High-resolution electrospray ionization source (ESI) LC/MS data were collected on an AB SCIEX Q-TOF 5600 high-resolution mass spectrometer at the National Instrumentation Center for Environmental Management (NICEM, Seoul, Republic of Korea).

### 3.2. Isolation and Identification of the Bacterial Strain Bacillus sp. AMD05

The strain, AMD05, was isolated from a mudflat sample collected from the intertidal mudflat on Anmyeon Island, Republic of Korea, using a sterilized 40 mL plastic tube. Various strain isolation media were applied for single-strain isolation, while AMD05 was isolated on a YEME-based agar medium (10 g/L of malt extract, 4 g/L of yeast extract, 4 g/L of glucose, and 18 g/L of agar) incubated at 25 °C for 7 days. The AMD05 strain was most closely related to *Bacillus velezensis* F-30 (97% identity, accession # MF988699) according to 16S rDNA sequence analysis (AMD05 16S rDNA GenBank deposit #OM319625).

### 3.3. Cultivation and Extraction

The spores of the bacterial strain *Bacillus* sp. AMD05 were inoculated into 50 mL of YEME liquid medium in a 125 mL flask. The culture was incubated at 200 rpm at 30 °C for two days. After incubation, 10 mL of the AMD05 liquid culture was inoculated into a 500 mL Erlenmeyer flask containing 200 mL of YEME medium and shaken at 170 rpm and 30 °C for two days. Then, 15 mL of the medium culture was transferred into 1 L of YEME medium in a 2.8 L Fernbach flask for four days at 170 rpm and 30 °C (24 ea × 1 L, total volume 24 L). The entire culture was extracted with 36 L of ethyl acetate (EtOAc). The EtOAc layer was separated using a separation funnel (capacity 3 L), and the residual water in the EtOAc layer was removed by adding anhydrous sodium sulfate. The extract was concentrated *in vacuo* to yield dry material. This procedure was repeated 3 times (72 L of culture in total) to yield extracted material.

### 3.4. Isolation of Succinyl Glyco-Oxydifficidin (1) and Succinyl Macrolactin O (2)

The dried extract material was dissolved in methanol (MeOH), adsorbed with Celite 545 (DaeJung Chmicals and Metals Co., Ltd., Siheung-si, Republic of Korea), and loaded onto a reversed-phase flash chromatography column (YMC C_18_ resin, 60 × 40 mm). Five MeOH/H_2_O concentrations (20%, 40%, 60%, 80%, and 100% of aqueous MeOH, each for 400 mL) were used for fractionation. Each fraction was analyzed by LC/MS, which indicated that **1** and **2** were eluted in the 80% aqueous MeOH fraction. Succinyl glyco-oxydifficidin and succinyl macrolactin O were further purified using semipreparative high-performance liquid chromatography (HPLC). The dried 80% MeOH fraction was subjected to reversed-phase HPLC (Kromasil C_18_ column, 5 μm, 10 × 250 mm) under a step gradient solvent system using 35–65% aqueous CH_3_CN from 0 to 30 min, which was continued with an isocratic 80% aqueous CH_3_CN method from 30 to 60 min (UV 230 nm detection, flow rate: 2 mL/min). Succinyl glyco-oxydifficidin was eluted at 32 min while **2** was eluted at 22 min. Succinyl glyco-oxydifficidin was further purified using a Kromasil C_18_ column at a retention time of 23 min (15 mg) under an isocratic condition (54% aqueous CH_3_CN). Succinyl macrolactin O was also purified by the same column at a retention of 33 min (10 mg) under an isocratic condition (39% aqueous CH_3_CN).

Succinyl glyco-oxydifficidin (**1**): Yellow powder, [α]_D_ = −19.2 (c 0.1, MeOH); UV (MeOH) λ_max_ (log ε) 234 (3.80), 274 (3.39), 284 (3.30) nm; IR (neat) ν_max_ 3414, 2926, 1725, 1424, 1266, 1166, 1081 cm^−1^; CD (MeOH) λ_max_ (log ε) 232 (−4.65), 272 (+4.15), 282 (+3.96) nm; for ^1^H and ^13^C NMR data, see Table 1, HRESIMS *m*/*z* 765.3814 [M + Na]^+^ (calcd for C_41_H_58_O_12_Na, 765.3826).

Succinyl macrolactin O (**2**): Yellow powder, [α]_D_ = −36.8 (c 0.1, MeOH); UV (MeOH) λ_max_ (log ε) 236 (3.86), 260 (3.70) nm; IR (neat) ν_max_ 3402, 2927, 1705, 1571, 1413, 1187, 1050 cm^−1^; CD (MeOH) λ_max_ (log ε) 233 (+5.26), 258 (−5.19) nm; for ^1^H and ^13^C NMR data, see Table 1, HRESIMS *m*/*z* 687.2977 [M+Na]^+^ (calcd for C_34_H_48_O_13_Na, 687.2992).

### 3.5. Determination of the Configuration of the Sugar in **1** and **2**

Succinyl glyco-oxydifficidin was hydrolyzed with 6 N HCl at 115 °C for 1 h to yield the free glucopyranose. After drying in vacuo, the acid hydrolysate was reacted with l-cysteine methyl ester hydrochloride and σ-tolyl isothiocyanate at 60 °C, each for 1 h. The authentic β-l-glucose and β-d-glucose were also reacted with l-cysteine methyl ester hydrochloride and σ-tolyl isothiocyanate at 60 °C, each for 1 h. The β-glucopyranose reaction product from **1** was co-injected with the products of the authentic β-l-glucose and β-d-glucose using LC/MS analysis (gradient solvent conditions: 10-100% aqueous CH_3_CN (0.1% formic acid) for 20 min). The reaction products of β-glucopyranose from **1** had the same retention time as the reaction product of authentic β-d-glucose (only one peak in the LC/MS spectrum). This method was also applied for succinyl macrolactin O (**2**), identifying β-d-glucose (Figure S28).

### 3.6. MTPA Esterification of Succinyl Glyco-Oxydifficidin (**1**)

Succinyl glyco-oxydifficidin was transferred to two 40 mL vials (2 mg of **1** in each vial) and dried completely under high vacuum overnight. A total of 1 mL of distilled anhydrous pyridine was added to each vial under argon gas. The mixtures were stirred at room temperature for approximately 5 min. Then, *R*- and *S*-α-methoxy-α-(trifluoromethyl) phenylacetyl chloride (MTPA-Cl) (50 μL) were added into one of the two vials. The reactions were terminated after 30 min by adding 50 μL of MeOH. The reaction mixtures were dried *in vacuo* and subjected to reversed-phase HPLC (Kromasil C_18_ column, 5 μm, 10 × 250 mm). An isocratic solvent system (94% aqueous CH_3_CN for 40 min, flow rate: 2 mL/min, detection: UV 230 nm) was used. The *S*-MTPA ester (**1a**) and the *R*-MTPA ester (**1b**) of **1** were both eluted at a retention time of 30 min. Low-resolution LC/MS analysis was carried out for 30 min using from 70% to 95% aqueous CH_3_CN (0.1% formic acid) with an IB chiral column. The Δ*δ_S-R_* values around the stereogenic centers were assigned by analyzing their ^1^H NMR and ^1^H-^1^H COSY NMR spectra (Figures S8–S11).

*S-*MTPA ester of **1** (**1a**): ^1^H NMR (800 MHz, CD_3_CN) *δ*_H_ 7.57–7.37 (14H overlapped), 6.59 (1H, dt, *J* = 16.5, 11.0), 6.48 (1H, m), 6.46 (1H, m), 6.35 (1H, d, *J* = 11.5), 6.32 (1H, d, *J* = 11.0), 6.21 (1H, t, *J* = 11.0), 6.05 (1H, m), 5.86 (1H, d, *J* = 11.0), 5.72 (1H, m), 5.67 (1H, m), 5.37 (1H, m), 5.06 (1H, m), 5.03 (2H, s), 4.96 (2H, m), 4.84 (1H, m), 4.57 (2H, s), 4.43 (1H, m), 4.37 (1H, d, *J* = 8.0), 4.17 (1H, m), 4.14 (1H, m), 4.00 (1H, t, *J* = 6.0), 3.78 (1H, m), 3.74 (1H, m), 3.52 (3H, s), 3.50 (3H, s), 3.41 (1H, d, *J* = 14.0), 3.40 (1H, m), 3.38 (3H, s), 3.08 (1H, d, *J* = 14.0), 2.73 (1H, m), 2.60 (1H, m), 2.54 (1H, m), 2.44 (1H, m), 2.42 (1H, m), 2.30 (1H, m), 2.11 (1H, m), 2.01 (1H, m), 1.93 (2H, m), 1.82 (1H, m), 1.79 (6H, s), 1.70 (1H, m), 1.34–1.24 (24H overlapped), 1.03 (3H, d, *J* = 7.0).

*R-*MTPA ester of **1** (**1b**): ^1^H NMR (800 MHz, CD_3_CN) *δ*_H_ 7.57–7.37 (14H overlapped), 6.58 (1H, dt, *J* = 16.5, 11.0), 6.47 (1H, m), 6.45 (1H, m), 6.40 (1H, d, *J* = 11.5), 6.33 (1H, d, *J* = 11.0), 6.19 (1H, t, *J* = 11.0), 6.03 (1H, t, *J* = 11.0), 5.86 (1H, d, *J* = 11.0), 5.69 (1H, m), 5.65 (1H, m), 5.37 (1H, m), 5.06 (1H, m), 5.05 (2H, s), 4.96 (1H, m), 4.83 (2H, m), 4.57 (2H, s), 4.43 (1H, m), 4.34 (1H, d, *J* = 8.0), 4.18 (1H, m), 4.10 (1H, m), 4.00 (1H, t, *J* = 6.0), 3.83 (1H, m), 3.75 (1H, m), 3.70 (1H, m), 3.60 (1H, m), 3.63 (3H, s), 3.47 (3H, s), 3.41 (1H, d, *J* = 14.0), 3.38 (3H, s), 3.07 (1H, d, *J* = 14.0), 2.69 (1H, m), 2.60 (1H, m), 2.50 (1H, m), 2.45 (1H, m), 2.39 (1H, q, *J* = 7.6, 7.2), 2.21 (1H, m), 2.11 (1H, t, *J* = 8.0), 1.98 (1H, m), 1.83 (2H, m), 1.82 (1H, m), 1.80 (3iiH, s), 1.79 (3H, s), 1.70 (1H, m), 1.34-1.24 (24H overlapped), 1.01 (3H, d, *J* = 7.0).

### 3.7. Conformational Search and DP4 Calculations

For the determination of the configurations of C-4, C-5, and C-21, **1c** (4*R*, 5*S*, and 21*S*), **1d** (4*R*, 5*S*, and 21*R*), **1e** (4*S*, 5*R*, and 21*S*), and **1f** (4*S*, 5*R*, and 21*R*) were generated by Avogadro 1.2.0. A conformational search for these diastereomers was performed by MacroModel with the Merck Molecular Force Field to find the stable conformers (with 10 kJ/mol energy limit) of the diastereomers: twelve conformers for **1c** (4*R*, 5*S*, and 21*S*), nine conformers for **1d** (4*R*, 5*S*, and 21*R*), six conformers for **1e** (4*S*, 5*R*, and 21*S*), and twenty-nine conformers for **1f** (4*S*, 5*R*, and 21*R*) (Table S1). The Boltzmann populations of the conformers were also calculated by MacroModel. Ground-state geometry optimization was performed by density functional theory (DFT) modeling of Turbomole X 4.3.2. All calculations were performed at the B3LYP/def-SV(P) level in the gas phase. This basis set, taken from the work [31], was used for all atoms. Calculated chemical shifts were calculated based on this equation: $\delta_{calc.}^{X}=\frac{\sigma^{0}-\sigma^{X}}{1-\sigma^{0}/10^{6}}$. $\delta_{calc.}^{X}$ is the calculated chemical shifts of nucleus x (e.g., ^1^H or ^13^C), while σ^X^ and σ^0^ are the calculated isotropic constants of nucleus x and tetramethylsilane (TMS) [33]. The calculated NMR chemical shifts of each conformer were averaged by the Boltzmann populations. By comparing these Boltzmann-population-averaged chemical shifts with the experimental chemical shifts of **1** (Table S2), the DP4 calculation result proposed **1c** (4*R*, 5*S*, and 21*S*) configurations with 100.0% probability using both carbon and proton data (71.0% probability using only the carbon data and 100.0% probability using only the proton data) (Figure S17).

### 3.8. Peptide Synthesis

Full-length Aβ_1-42_ with a C-terminal cysteine, Aβ_1-42_-cys, was synthesized via solid-phase peptide synthesis, as previously reported. Fluorescent Aβ_1-42_ was synthesized with the conjugation of Flamma-552 carboxylic acid on N-terminus [26,32].

### 3.9. Aβ Aggregation Assay Plate Preparation

A maleimide-activated microplate was used to immobilize the peptide. The bovine serum albumin coated on the maleimide-activated microplate was removed after washing the peptide three times with 200 µL of wash buffer (0.1 M sodium phosphate, 0.15 M sodium chloride, 0.05% Tween-20; pH 7.2) in each well on the plate. A full-length Aβ_1-42_-cys solution (50 µg/mL, 5% DMSO) was made in binding buffer (0.1 M sodium phosphate, 0.15 M sodium chloride, 10mM EDTA; pH 7.2). A total of 100 µL of 10 µM peptide solution was added to each well and reacted with maleimide for 24 h at RT. After peptide immobilization, the unbounded peptides were washed three times with 200 µL of wash buffer. To inactivate additional maleimide groups, 200 µL of cysteine solution was added to each well and incubated at RT for 1 h. After cysteine capping, all the wells in the plate were washed three times with 200 µL of wash buffer.

### 3.10. Aβ Aggregation Assay

To examine the inhibition effect of succinyl glyco-oxydifficidin and succinyl macrolactin O, 50 µL of succinyl macrolactin and succinyl glyco-oxydifficidin was prepared in binding buffer (1% DMSO), and 50 µL of Flmma 552-labeled full-length Aβ_1-42_ peptide solution (20 µM) was prepared in binding buffer (1% DMSO). Each solution was made with two concentrations: 5 µM and 50 µM. The solution containing two compounds and Flamma-552-labeled full-length Aβ_1-42_ peptide was added to the plate and incubated for 24 h at RT. After the incubation, all the wells were washed three times with 200 µL of wash buffer. For the comparison, we only added 100 µL of Flamma-labeled full-length Aβ_1-42_ peptide solution (10 µM) on the other wells and incubated them for 24 h at RT. The intensity of the dug-treated well and control well was measured by the microplate reader.

### 3.11. Aβ Disociationn Assay

Before treating succinyl glyco-oxydifficidin and succinyl macrolactin O, 100 μL of fluorescent Aβ_1-42_ peptide solution (10 μM) was added to the wells and incubated for 8 h at 37 °C. After the incubation, all the wells were washed three times with 200 μL of the wash buffer. Succinyl macrolactin (5, 50 µM) and succinyl glyco-oxydifficidin (5, 50 µM) were prepared in a binding buffer (1% DMSO). EPPS and necrostatin-1 (5 µM each) were prepared as positive controls. We added 100 μL of each compound solution to the wells and incubated them for 24 h at RT. After the incubation, all the wells were washed three times with 200 μL of the wash buffer, and 100 μL of binding buffer was added to each well prior to reading. Fluorescent scanning was carried out with a microplate reader (555/580 nm, ex/em).

### 3.12. Docking Model Generation

The 500 three-dimensional conformers were generated using RDKit (https://www.rdkit.org/, accessed on 15 December 2022) with 0.2 Å RMSD threshold for succinyl glyco-oxydifficidin and succinyl macrolactin O, respectively. Each conformer was used to define potential binding sites by applying global docking with PatchDock program [34] without a predefined binding region. The U-shaped Aβ_1-42_ (PDB ID: 2BEG) was used as a receptor structure. The docking search space for the receptor was confined to edge strands of β-sheets to reflect the experimental results (Figure 7). The top 10 docking models for each conformer were retrieved to infer binding site information (center_x, center_y, and center_z parameters) for the subsequent docking refinement by Autodock Vina [35]. The final docking model was selected based on the Autodock Vina score.

### 3.13. Statistical Data Evaluation

All results were given as a mean ± standard error of the mean (SEM). All data were analyzed through GraphPad Prism 9.0 software and compared using One-way ANOVA analysis followed by Bonferroni’s post-hoc comparison. * *p* < 0.05, ** *p* < 0.01, *** *p* < 0.001, **** *p* < 0.0001.

## 4. Conclusions

Succinyl glyco-oxydifficidin (**1**), a new glycosylated and succinylated member of the difficidin family, was discovered in an intertidal mudflat-derived *Bacillus* sp. AMD05. Compared its structure with that of oxydifficidin, succinyl glyco-oxydifficidin (**1**) was modified through glycosylation and succinylation, along with a double-bond migration from C-6 to C-7. Although difficidin was discovered more than 35 years ago in 1986, its absolute configuration had remained undetermined until now. By using combinational tools of spectroscopic analysis, the modified Mosher’s method, and DP4 computational calculation, we elucidated the absolute configuration of succinyl glyco-oxydifficidin (**1**) for the first time among the difficidin family compounds. Interestingly, succinyl macrolactin O (**2**) is the only compound in the macrolactin family to bear both glucose and succinyl acid.

Furthermore, succinyl glyco-oxydifficidin (**1**) inhibited/dissociated Alzheimer-disease-related Aβ aggregation and succinyl macrolactin O (**2**) inhibited Aβ aggregation, indicating their therapeutic potential to regulate Aβ aggregation. Even though the difficidin and macrolactin families were discovered in the 1980s with anti-microbial bioactivity [11,23], their Aβ-regulating activities were observed for the first time here. Succination and glycosylation may distribute to bioactive modification. Our discovery of the new glycosylated and succinylated macrocyclic lactones with Aβ-regulating activity from marine *Bacillus* sp. highlights that marine bacteria are prolific sources of natural products diversified by glycosylation, an important biological process for changing the structures and bioactivity of compounds.

## Figures and Tables

**Table 1:** NMR data for **1** and **2** in CD_3_OD.

	1		2		
C/H	*δ*_C_, Type	*δ*_H_, Mult (*J* in Hz)	C/H	*δ*_C_, Type	*δ*_H_, Mult (*J* in Hz)
1	174.03, C		1	167.97, C	
2	47.03, CH_2_	3.059, d (14.5)	2	117.83, CH	5.544, d (11.0)
		3.444, d (14.5)	3	145.37, CH	6.628, d (11.0)
3	148.67, C		4	130.23, CH	7.229. dd (15.0, 11.0)
4	41.19, CH	2.369, m	5	141.73, CH	6.214, ddd (15.0, 8.0, 6.0)
5	86.94, CH	3.693, m	6	40.97, CH_2_	2.560, m
6	32.03, CH_2_	2.512, m			2.456, m
		2.732, m	7	78.73, CH	4.370, dd (13.0, 7.0)
7	128.61, CH	5.639, td (11.0, 4.0)	8	134.17, CH	5.636, dd (15.0, 7.0)
8	128.72, CH	6.440, t (12.5)	9	129.45, CH	6.581, dd (15.0, 11.0)
9	123.86, CH	6.162, m	10	131.71, CH	6.146, t (11.0)
10	130.46, CH	5.998, t (11.0)	11	128.85, CH	5.545, m
11	126.55, CH	6.439, t (12.5)	12	35.85, CH_2_	2.463, m
12	135.80, CH	5.707, m			2.373, dt (14.0, 7.0)
13	29.18, CH_2_	1.945, m	13	68.66, CH	4.118, m
		2.311, m	14	49.42, CH_2_	2.580, d (6.5)
14	34.10, CH_2_	1.803, m			2.555, d (6.5)
		1.739, m	15	211.82, C	
15	68.14, CH	4.680, dd (9.0, 6.0)	16	44.40, CH_2_	2.461, m
16	140.83, C		17	27.95, CH_2_	2.197, m
17	123.89, CH	6.232, d (11.0)	18	130.30, CH	5.411, m
18	126.58, CH	6.208, d (11.0)	19	132.01, CH	5.411, m
19	126.47, CH	5.257, m	20	33.00, CH_2_	2.051, ddd (17.0, 12.0, 5.5)
20	32.29, CH_2_	2.403, m			1.959, dt (13.0, 7.0)
		2.601, m	21	26.19, CH_2_	1.428, m
21	75.89, CH	4.802, m			1.393, m
22	33.45, CH_2_	1.703, m	22	36.29, CH_2_	1.630, m
		1.817, m			1.531, m
23	36.75, CH_2_	2.102, t (8.0)	23	71.75, CH	5.001, m
24	139.27, C		24	20.26, CH_3_	1.232, m
25	127.37, CH	5.861, broad d (11.0)	25	101.37, CH	4.309, d (8.0)
26	134.48, CH	6.584, dtd (14.5, 10.5, 4.0)	26	75.07, CH	3.239, d (8.5)
27	115.53, CH_2_	4.969, dd (10.0, 2.0)	27	78.00, CH	3.328, dd (18.0, 9.0)
		5.071, dd (17.0, 2.0)	28	71.78, CH	3.300, m
28	105.91, CH	4.285, d (8.0)	29	75.28, CH	3.402, ddd (9.0, 6.0, 2.0)
29	75.50, CH	3.188, dd (9.0, 8.0)	30	64.94, CH_2_	4.423, dd (12.0, 2.0)
30	78.09, CH	3.315, m			4.250, dd (12.0, 2.0)
31	71.92, CH	3.267, d (9.0)	31	174.21, C	
32	75.19, CH	3.420, ddd (9.0, 6.5, 2.5)	32	29.97, CH_2_	2.605, t (6.5)
33	64.93, CH_2_	4.405, dd (12.0, 2.0)	33	30.17, CH_2_	2.654, t (6.5)
		4.220, dd (12.0, 6.5)	34	176.15, C	
34	174.37, C				
35	30.90, CH_2_	2.546, m			
36	30.64, CH_2_	2.596, m			
37	177.20, C				
38	113.99, CH_2_	5.026, s 5.046, s			
39	17.40, CH_3_	0.979, d (7.0)			
40	17.76, CH_3_	1.802, s			
41	16.62, CH_3_	1.779, s			

^1^H 800 MHz, ^13^C 200 MHz.

## Data Availability

The original contributions presented in the study are included in the article/Supplementary Material; further inquiries can be directed to the corresponding author.

## Supplementary Materials

The following supporting information can be downloaded at: https://www.mdpi.com/article/10.3390/md21020067/s1, Figure S1. ^1^H NMR spectrum of **1** at 800 MHz in CD_3_OD; Figure S2. ^13^C NMR spectrum of **1** at 800 MHz in CD_3_OD; Figure S3. COSY spectrum of **1** at 800 MHz in CD_3_OD; Figure S4. HSQC spectrum of **1** at 800 MHz in CD_3_OD; Figure S5. HMBC spectrum of **1** at 800 MHz in CD_3_OD; Figure S6. ROESY spectrum of **1** at 800 MHz in CD_3_OD; Figure S7. HETLOC spectrum of **1** at 800 MHz in CD_3_OD; Figure S8. H-NMR of *S*-MTPA ester of **1** at 800 MHz in CD_3_OD; Figure S9. COSY NMR of *S*-MTPA ester of **1** at 800 MHz in CD_3_OD; Figure S10. H-NMR of *R*-MTPA ester of **1** at 800 MHz in CD_3_OD; Figure S11. COSY NMR of *R*-MTPA ester of **1** at 800 MHz in CD_3_OD; Figure S12. UV spectrum of **1;** Figure S13. CD spectrum of **1**; Figure S14. IR spectrum of **1**; Figure S15. HR-ESI-MS spectrum of 1; Figure S16. LC/MS analysis of β-glucopyranose reaction product from **1** coinjecting with each authentic β-L-glucose reaction product and authentic β-D-glucose reaction product; Figure S17. Statistical comparison of the calculated and experimental chemical shifts by the tool of DP4 calculation; Figure S18. ^1^H NMR spectrum of **2** at 800 MHz in CD_3_OD; Figure S19. ^13^C NMR spectrum of **2** at 800 MHz in CD_3_OD; Figure S20. COSY spectrum of **2** at 800 MHz in CD_3_OD; Figure S21. HSQC spectrum of **2** at 800 MHz in CD_3_OD; Figure S22. HMBC spectrum of **2** at 800 MHz in CD_3_OD; Figure S23. ROESY spectrum of **2** at 800 MHz in CD_3_OD; Figure S24. UV spectrum of **2**; Figure S25. CD spectrum of **2**; Figure S26. IR spectrum of **2**; Figure S27. HR-ESI-MS spectrum of 2; Figure S28. LC/MS analysis of β-glucopyranose reaction product from **2** coinjecting with each authentic β-L-glucose reaction product and authentic β-D-glucose reaction product; Table S1. The major conformers (with 10 kJ/mol energy limit) of diastereomers **1c** (4*R*, 5*S*, and 21*S*), **1d** (4*R*, 5*S*, and 21*R*), **1e** (4*S*, 5*R*, and 21*S*) and **1f** (4*S*, 5*R*, and 21*R*) of **1** without succinate group identified by conformational searches in MMFF94 force field using MacroModel and their Boltzmann population; Table S2. Experimental and calculated chemical shift (*δ*_H_) of diastereomers **1c** (4*R*, 5*S*, and 21*S*), **1d** (4*R*, 5*S*, and 21*R*), **1e** (4*S*, 5*R*, and 21*S*) and **1f** (4*S*, 5*R*, and 21*R*); Table S3. Cartesian coordinates for all atoms in the lowest energy optimized conformer **1**c (4*R*, 5*S*, 21*S*); Table S4. Antibacterial assay of **1** and **2**; Table S5. Antifungal assay of **1** and **2**; Table S6. Antituberculosis assay of **1** and **2**.
