# Supplementary material for: Glycosylated and Succinylated Macrocyclic Lactones with Amyloid-β-Aggregation-Regulating Activity from a Marine Bacillus sp
Source: Mar Drugs. 2023 Jan 19;21(2):67. doi: 10.3390/md21020067 (PMC9962899; doi:10.3390/md21020067)
Supplement: Supplementary file 1 [file marinedrugs-21-00067-s001.zip › marinedrugs-2096202-supplementary.pdf]

Supplementary Material

Glycosylated and succinylated macrocyclic lactones with amyloid-β-aggregation-regulating activity from a marine *Bacillus* sp.

Jinsheng Cui[1], Suhyun Ye[2], Daniel Shin[1], Illhwan Cho[2], Hye Yun Kim[2], Yun Kwon[3], Keunwan Park[4], Sang-Jip Nam[5], YoungSoo Kim[2,*], and Dong-Chan Oh[1,*]

[1]*Natural Products Research Institute, College of Pharmacy, Seoul National University, Seoul 08826, Republic of Korea;*

[2]*Department of Pharmacy and Yonsei Institute of Pharmaceutical Sciences, College of Pharmacy, Yonsei University, Incheon 21983, Republic of Korea;*

[3]*Research Institute of Pharmaceutical Science, College of Pharmacy, Kyungpook National University, Daegu 41566, Republic of Korea*

[4]*Natural Product Informatics Research Center, Korea Institute of Science and Technology, Gangwon-do 25451, Republic of Korea*

[5]*Department of Chemistry and Nanoscience, Ewha Womans University, Seoul 03760, Republic of Korea*

*** Correspondence**:

YoungSoo Kim

y.kim@yonsei.ac.kr

Dong-Chan Oh

dongchanoh@snu.ac.kr

Table of Contents

Figure S1. ^{1}H NMR spectrum of **1** at 800 MHz in CD_3OD.
Figure S2. ^{13}C NMR spectrum of **1** at 800 MHz in CD_3OD.
Figure S3. COSY spectrum of **1** at 800 MHz in CD_3OD.
Figure S4. HSQC spectrum of **1** at 800 MHz in CD_3OD.
Figure S5. HMBC spectrum of **1** at 800 MHz in CD_3OD.
Figure S6. ROESY spectrum of **1** at 800 MHz in CD_3OD.
Figure S7. HETLOC spectrum of **1** at 800 MHz in CD_3OD.
Figure S8. H-NMR of *S*-MTPA ester of **1** at 800 MHz in CD_3OD.
Figure S9. COSY NMR of *S*-MTPA ester of **1** at 800 MHz in CD_3OD.
Figure S10. H-NMR of *R*-MTPA ester of **1** at 800 MHz in CD_3OD.
Figure S11. COSY NMR of *R*-MTPA ester of **1** at 800 MHz in CD_3OD.
Figure S12. UV spectrum of **1.**
Figure S13. CD spectrum of **1**.
Figure S14. IR spectrum of **1**.
Figure S15. HR-ESI-MS spectrum of **1**.
Figure S16. LC/MS analysis of β-glucopyranose reaction product from **1** coinjecting with each authentic β-L-glucose reaction product and authentic β-D-glucose reaction product.
Table S1. The major conformers (with 10 kJ/mol energy limit) of diastereomers **1c** (4*R*, 5*S*, and 21*R*), **1d** (4*R*, 5*S*, and 21*S*), **1e** (4*S*, 5*R*, and 21*R*) and **1f** (4*S*, 5*R*, and 21*S*) of **1** identified by conformational searches in MMFF94 force field using MacroModel and their Boltzmann population.
Table S2. Experimental and calculated chemical shift (δ_H) of diastereomers **1c** (4*R*, 5*S*, and 21*R*), **1d** (4*R*, 5*S*, and 21*S*), **1e** (4*S*, 5*R*, and 21*R*) and **1f** (4*S*, 5*R*, and 21*S*) of **1**.
Figure S17. Statistical comparison of the calculated and experimental chemical shifts by the tool of DP4 calculation.
Figure S18. ^{1}H NMR spectrum of **2** at 800 MHz in CD_3OD.
Figure S19. ^{13}C NMR spectrum of **2** at 800 MHz in CD_3OD.
Figure S20. COSY spectrum of **2** at 800 MHz in CD_3OD.
Figure S21. HSQC spectrum of **2** at 800 MHz in CD_3OD.
Figure S22. HMBC spectrum of **2** at 800 MHz in CD_3OD.
Figure S23. ROESY spectrum of **2** at 800 MHz in CD_3OD.
Figure S24. UV spectrum of **2**.
Figure S25. CD spectrum of **2**.
Figure S26. IR spectrum of **2**.
Figure S27. HR-ESI-MS spectrum of **2**.
Figure S28. LC/MS analysis of β-glucopyranose reaction product from **2** coinjecting with each authentic β-L-glucose reaction product and authentic β-D-glucose reaction product.

Figure S1. ^{1}H NMR spectrum of **1** at 800 MHz in CD_3OD.

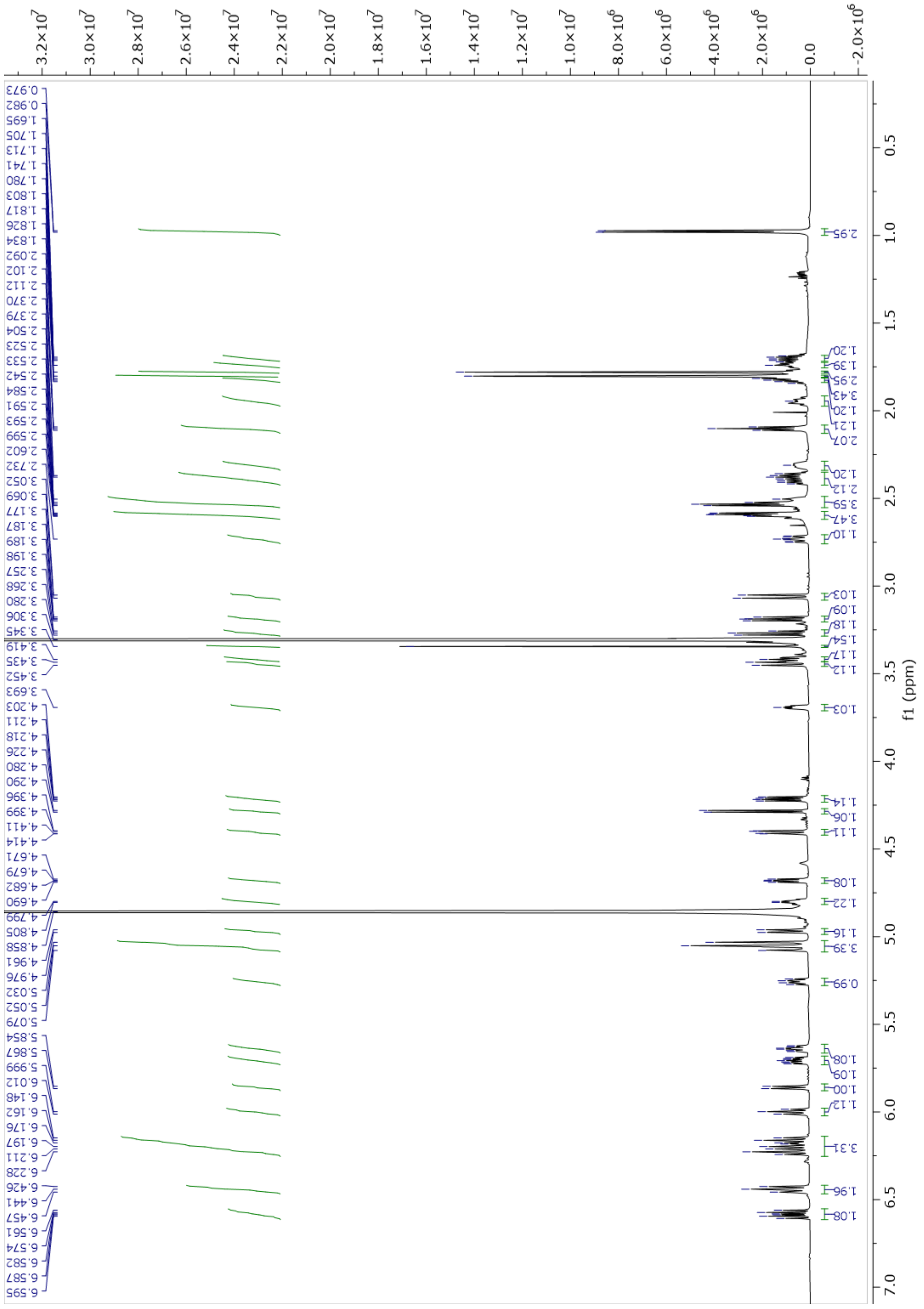

Figure S2. ^{13}C NMR spectrum of **1** at 800 MHz in CD_3OD.

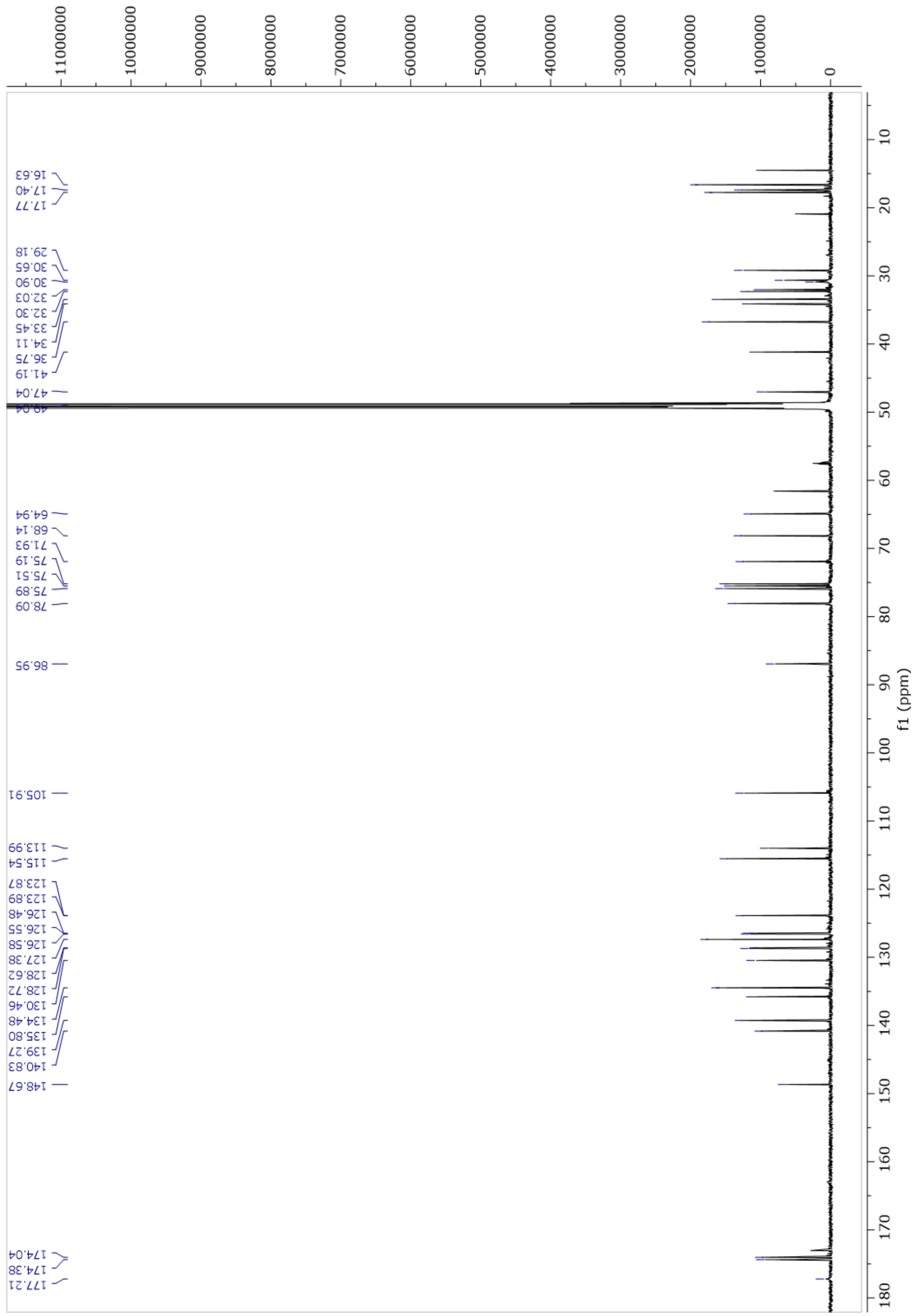

Figure S3. COSY spectrum of **1** at 800 MHz in CD_3OD.

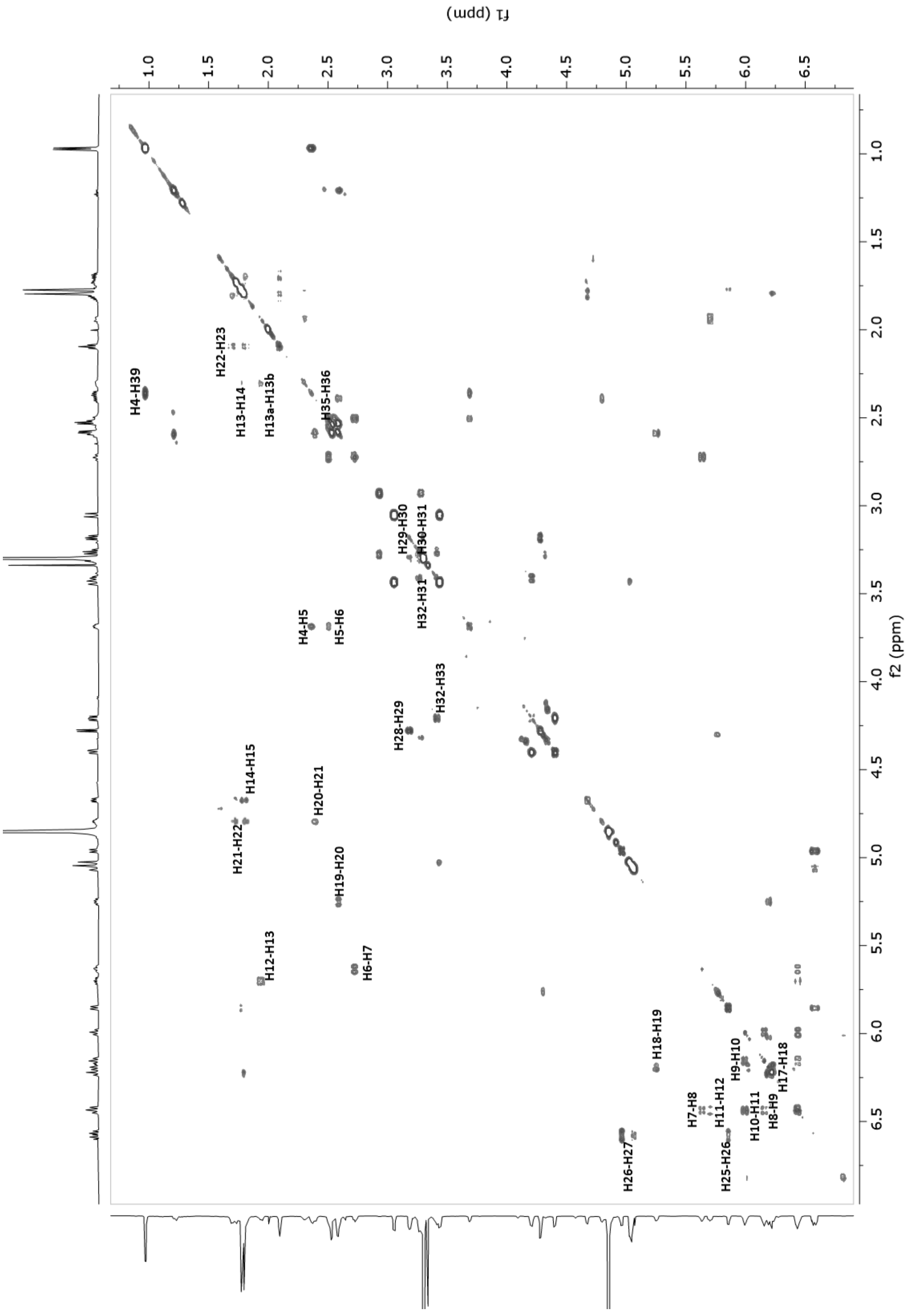

Figure S4. HSQC spectrum of **1** at 800 MHz in CD_3OD.

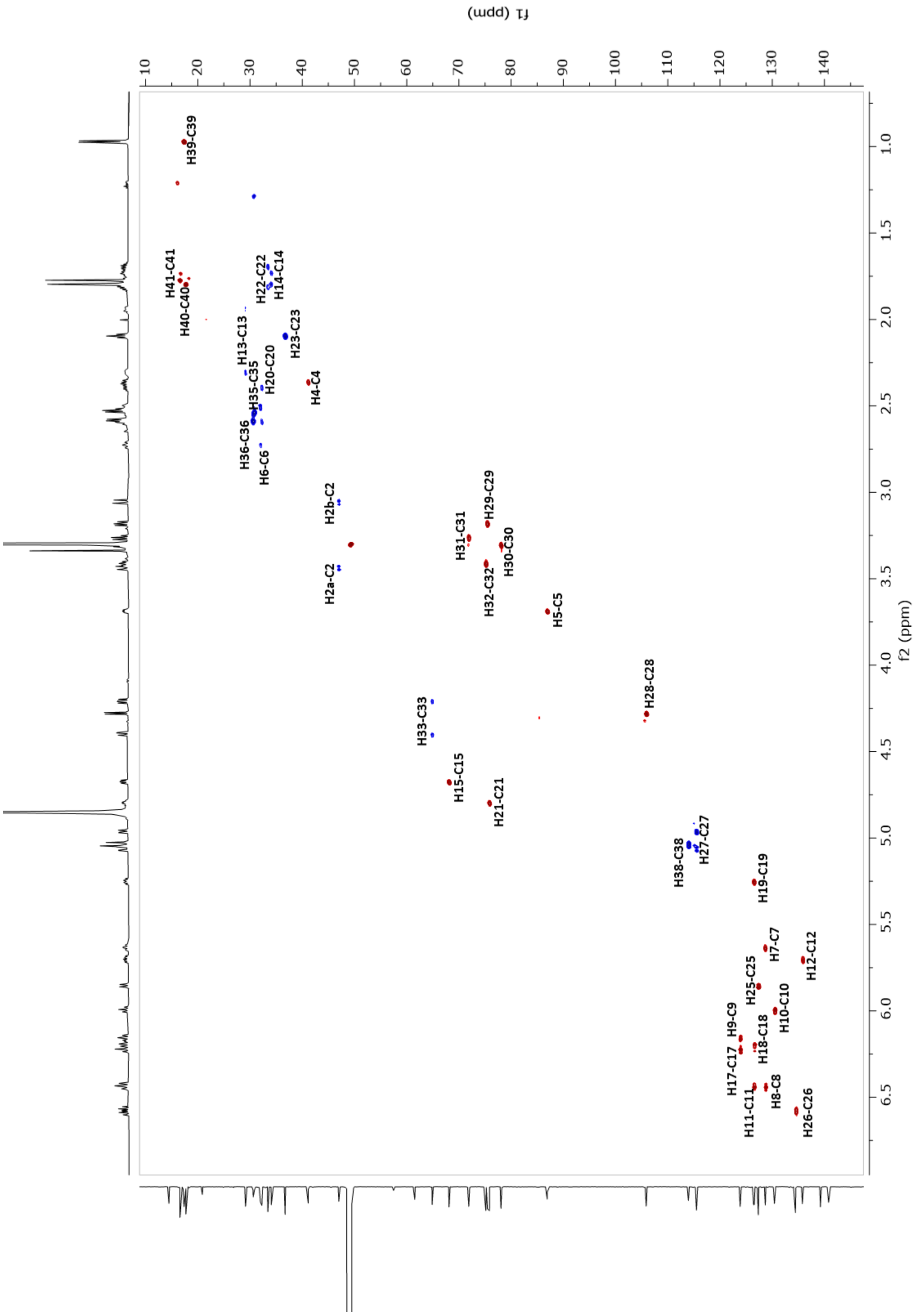

Figure S5. HMBC spectrum of **1** at 800 MHz in CD_3OD.

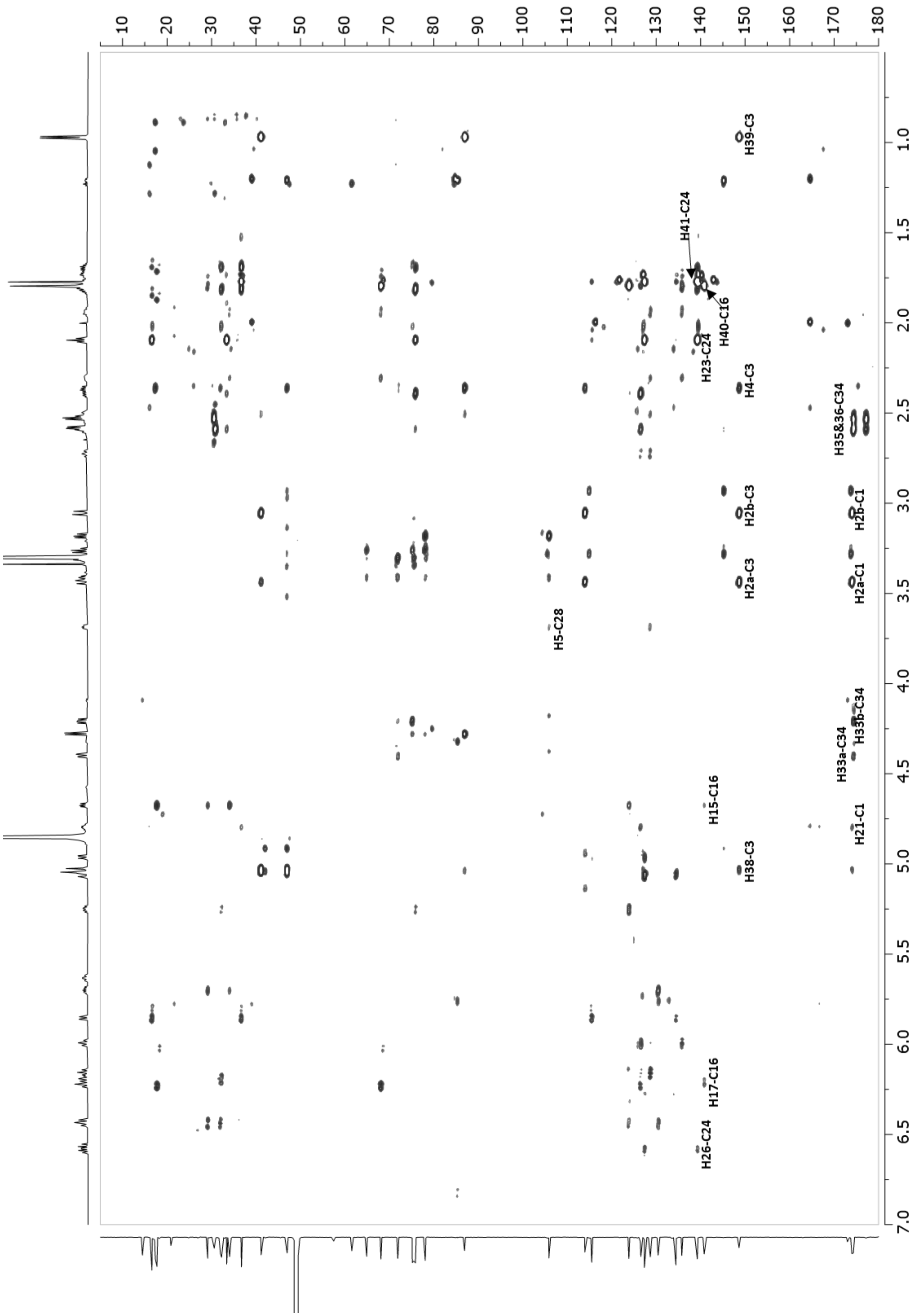

Figure S6. ROESY spectrum of **1** at 800 MHz in CD_3OD

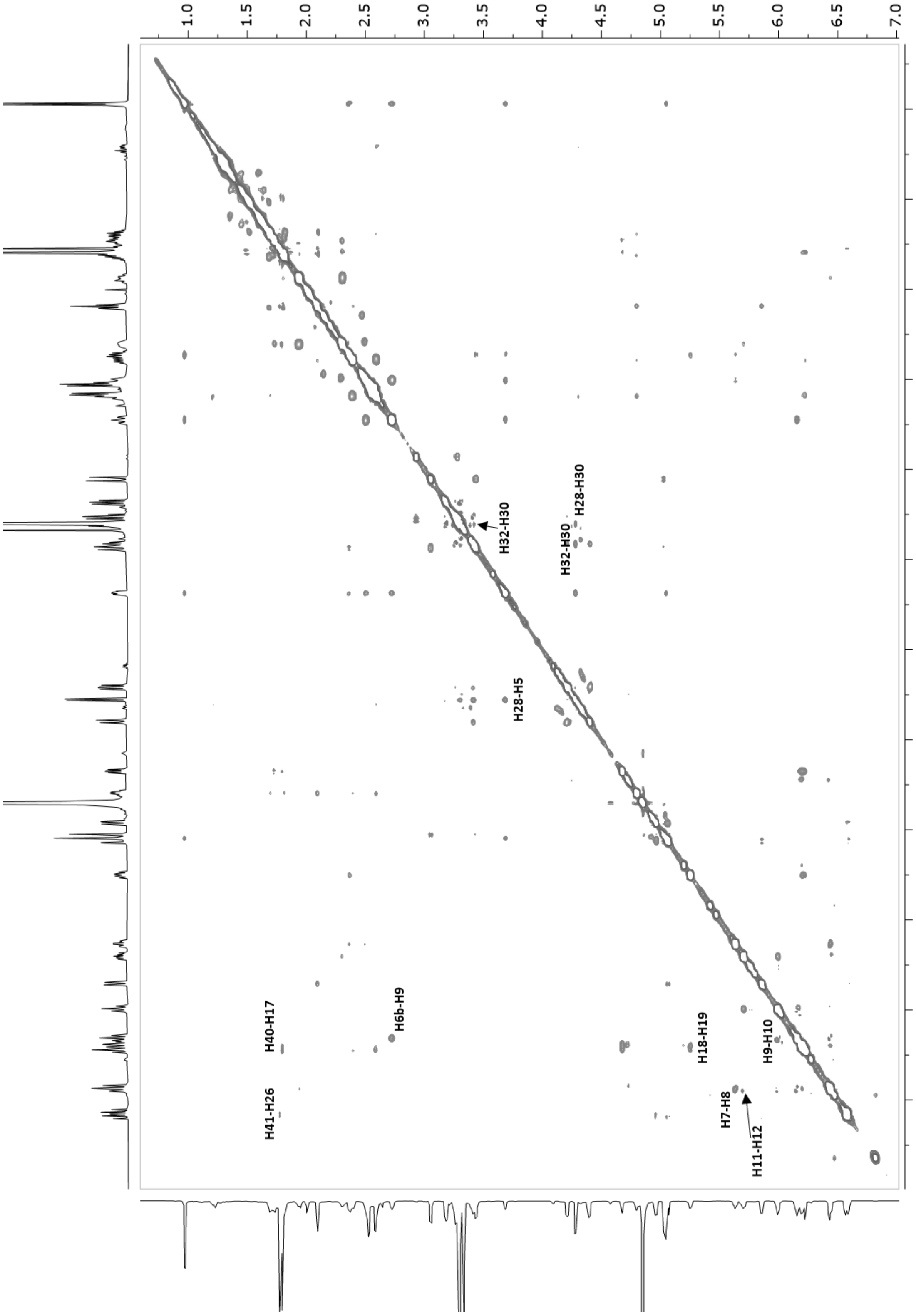

Figure S7. HETLOC spectrum of **1** at 800 MHz in CD_3OD.

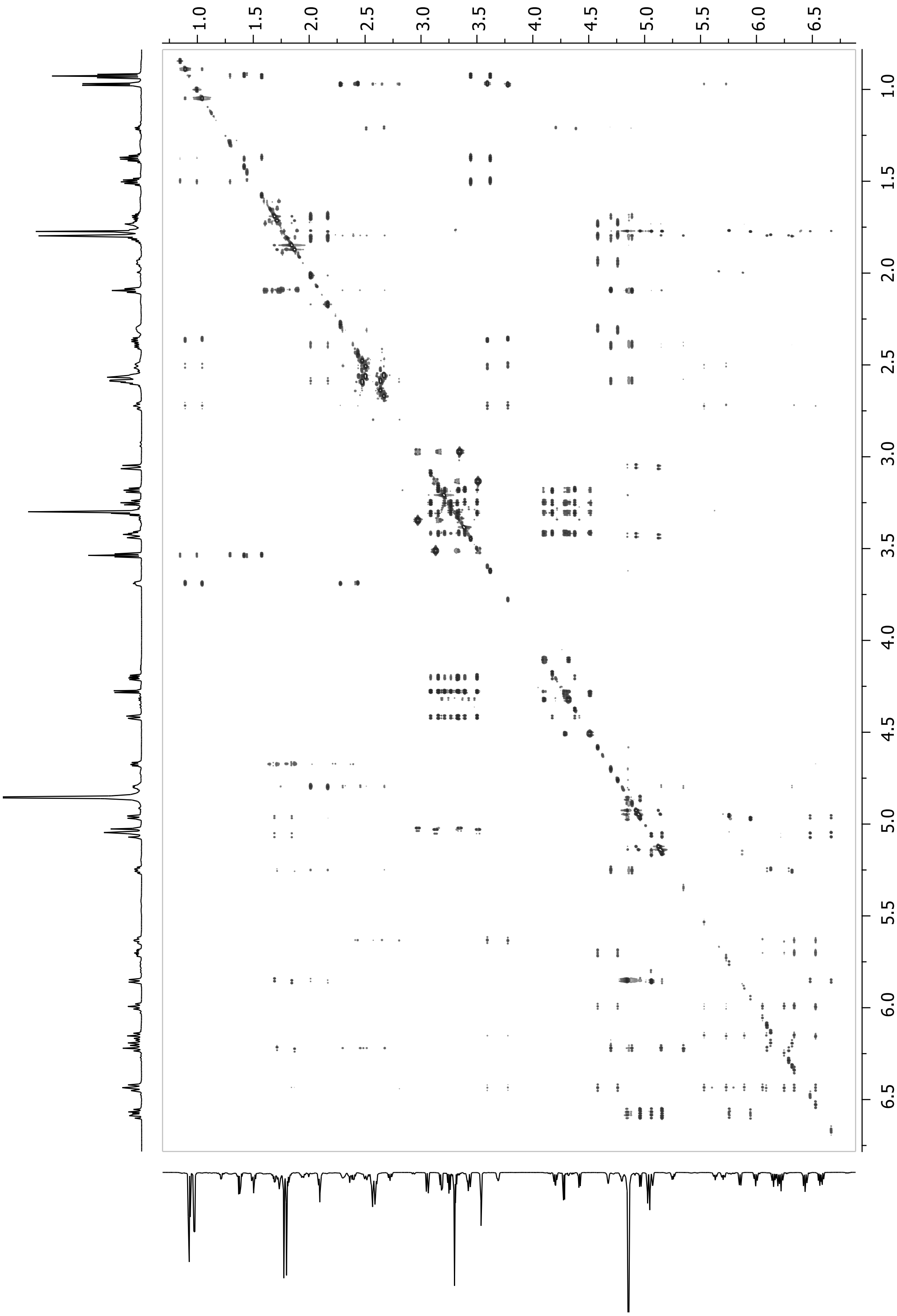

Figure S8. H-NMR of *S*-MTPA ester of **1** at 800 MHz in CD_3OD.

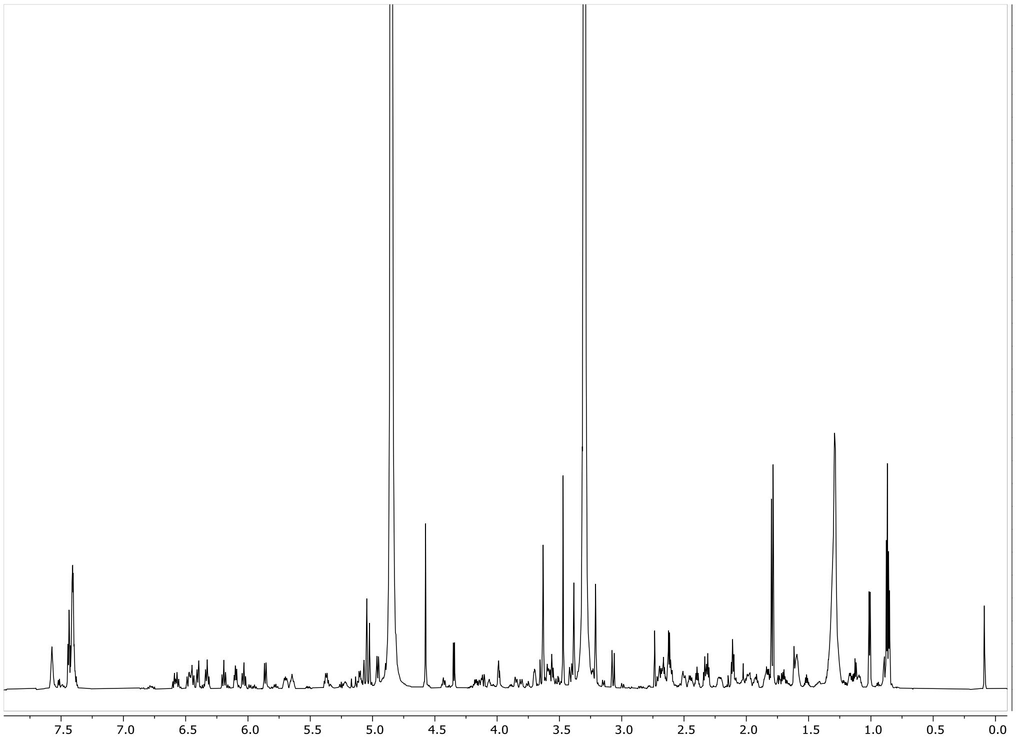

Figure S9. COSY NMR of *S*-MTPA ester of **1** at 800 MHz in CD_3OD.

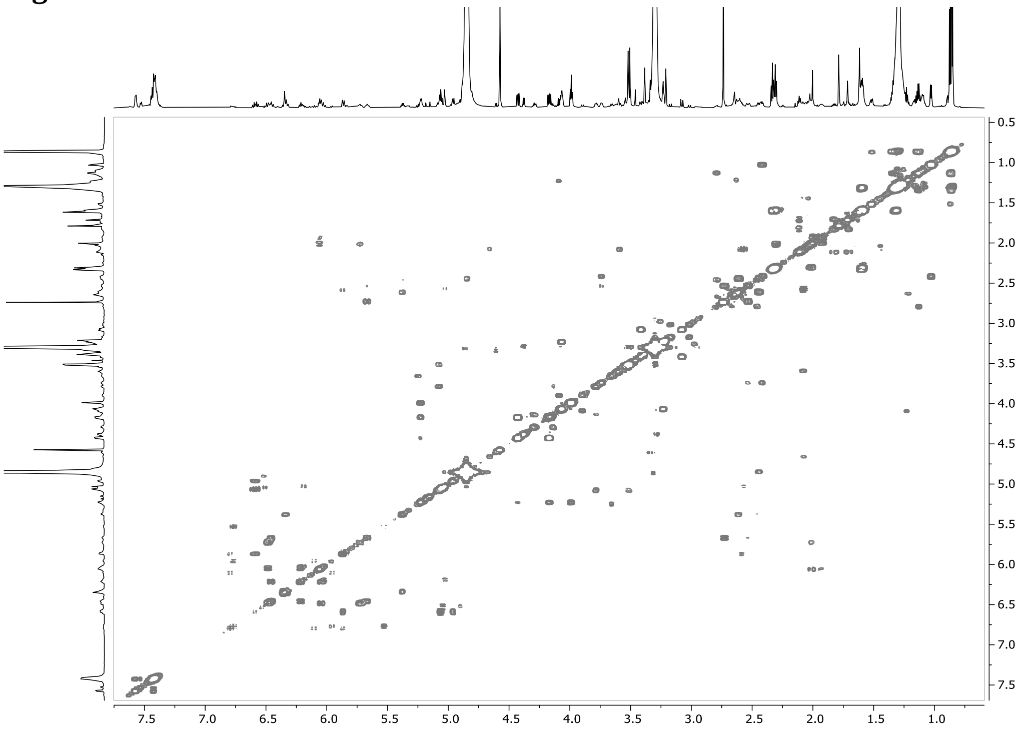

Figure S10. H-NMR of *R*-MTPA ester of **1** at 800 MHz in CD_3OD.

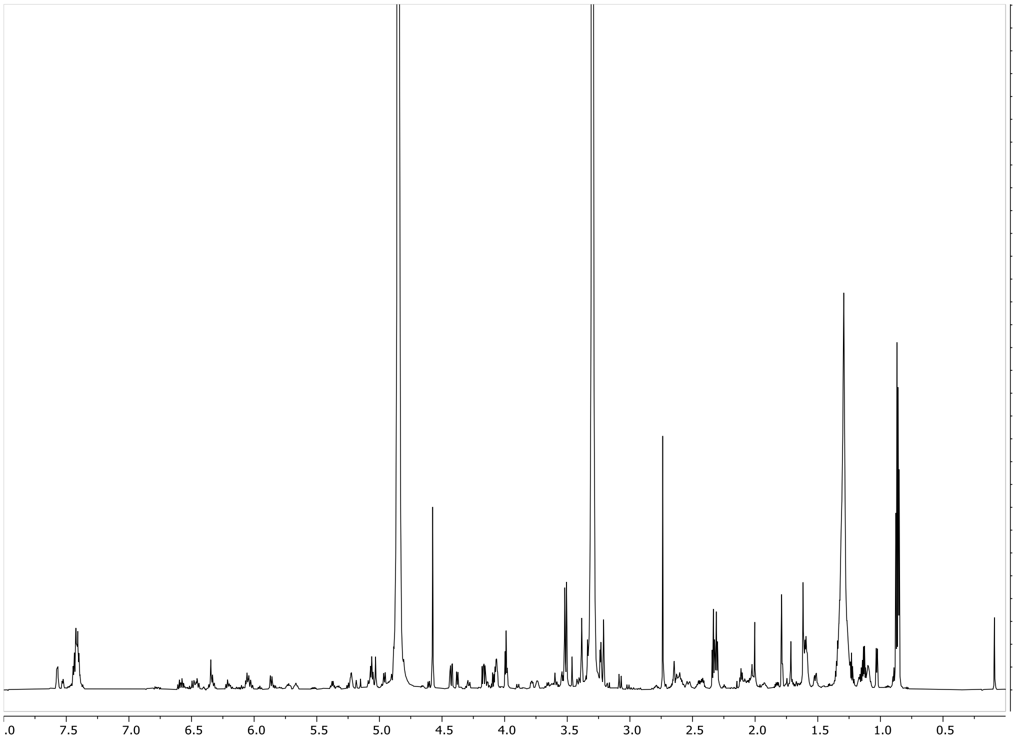

Figure S11. COSY NMR of *R*-MTPA ester of **1** at 800 MHz in CD_3OD.

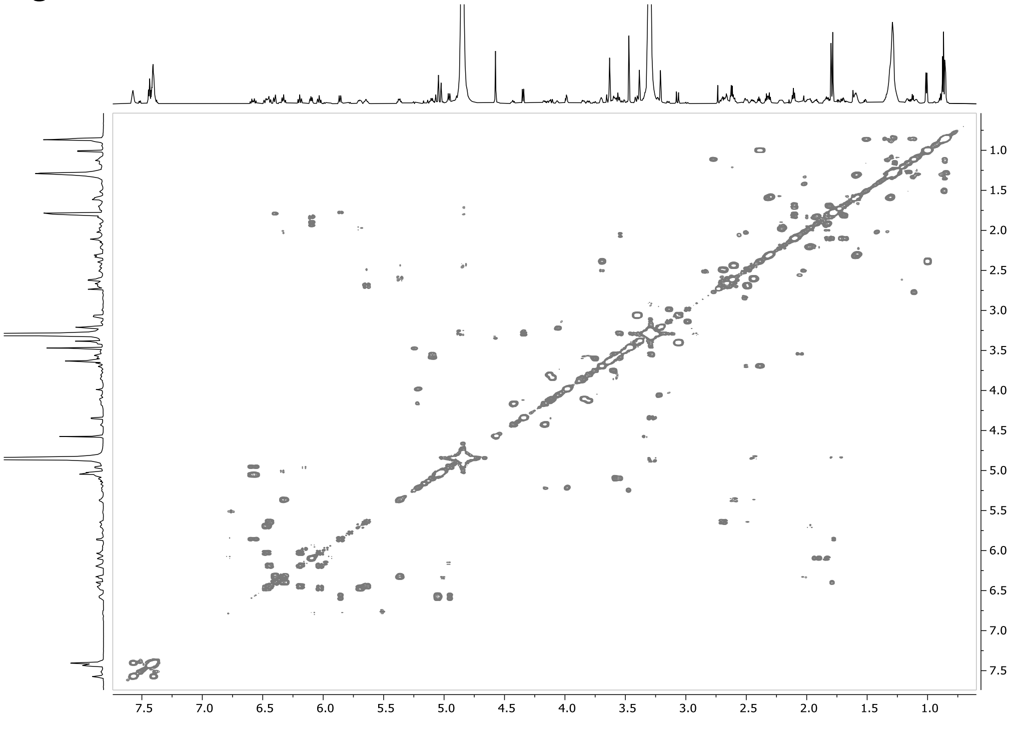

Figure S12. UV spectrum of **1.**

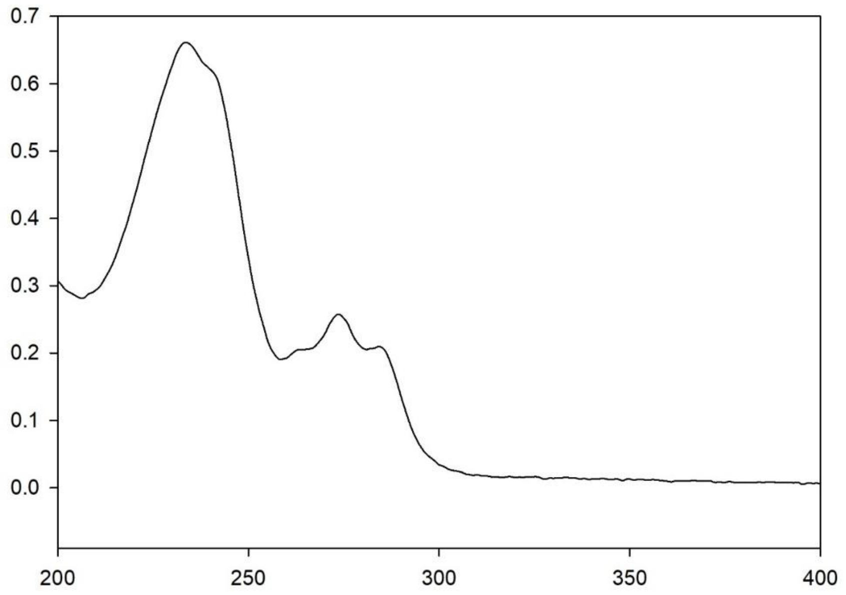

Figure S13. CD spectrum of **1**.

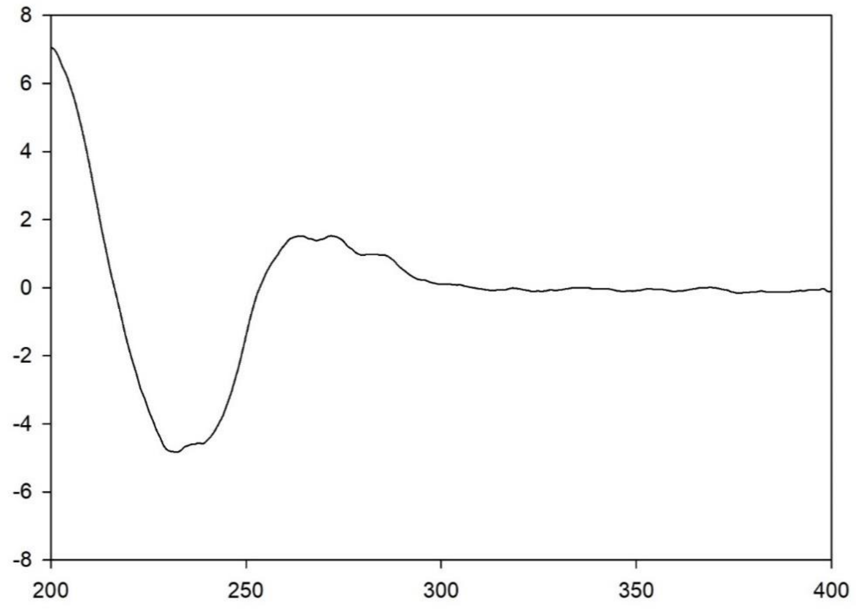

Figure S14. IR spectrum of **1**.

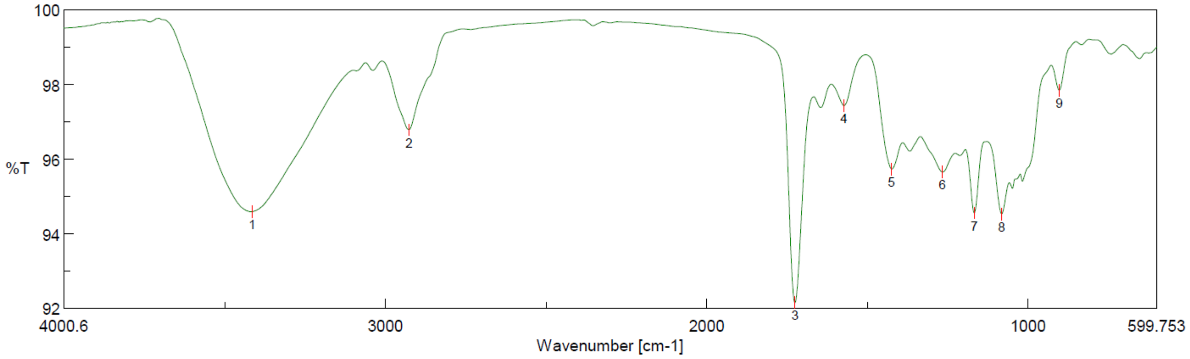

[Data Information]

Creation Date	2021-06-03 오후 3:44
Data array type	Linear data array
Horizontal	Wavenumber [cm-1]
Vertical	%T
Start	599.753 cm-1
End	4000.6 cm-1
Data pitch	0.964233 cm-1
Data points	3528

[Result of Peak Picking]

No.	Position	Intensity	No.	Position	Intensity	No.	Position	Intensity	No.	Position	Intensity
1	3414.35	94.5959	2	2926.45	96.7937	3	1725.01	92.1568	4	1572.66	97.4391
5	1424.17	95.7376	6	1266.04	95.6597	7	1166.72	94.5634	8	1081.87	94.5362
9	902.523	97.8543									

Figure S15. HR-ESI-MS spectrum of **1**.

Spectrum from AMD05_MW742.wiff (sample 1) - AMD05_MW742, Experiment 1, +TOF MS (100 - 2000) from 0.460 min

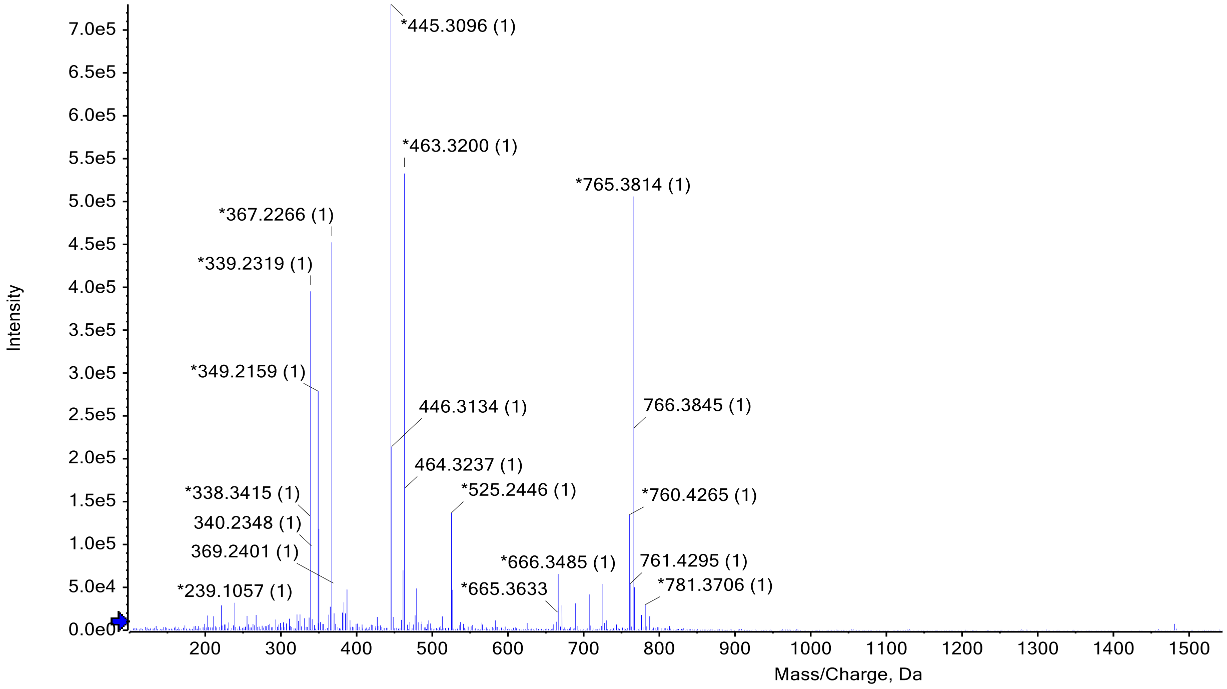

Figure S16. LC/MS analysis of β-glucopyranose reaction product from **1** coinjecting with each authentic β-L-glucose reaction product and authentic β-D-glucose reaction product.

β-glucopyranose reaction product of **1** + authentic β-L-glucose reaction product coinjection:

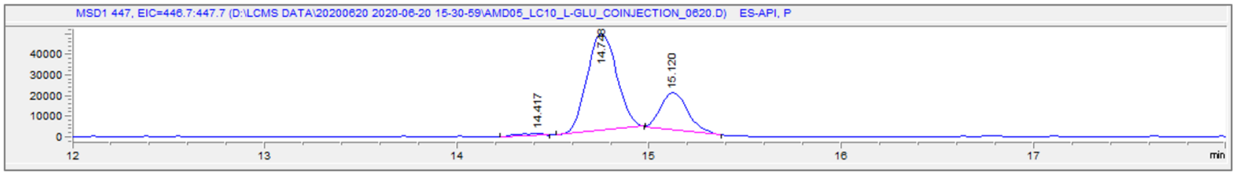

β-glucopyranose reaction product of **1** + authentic β-D-glucose reaction product coinjection:

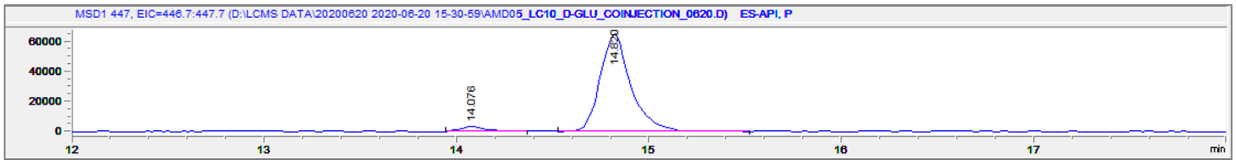

Table S1. The major conformers (with 10 kJ/mol energy limit) of diastereomers **1c** (4*R*, 5*S*, and 21*R*), **1d** (4*R*, 5*S*, and 21*S*), **1e** (4*S*, 5*R*, and 21*R*) and **1f** (4*S*, 5*R*, and 21*S*) of **1** identified by conformational searches in MMFF94 force field using MacroModel and their Boltzmann population.

Conformers	Boltzmann population (%)	Conformers	Boltzmann population (%)
Diastereomers **1c**_1	34.915	Diastereomers **1f**_2	15.968
Diastereomers **1c**_2	21.160	Diastereomers **1f**_3	11.282
Diastereomers **1c**_3	11.935	Diastereomers **1f**_4	9.143
Diastereomers **1c**_4	11.660	Diastereomers **1f**_5	7.836
Diastereomers **1c**_5	7.766	Diastereomers **1f**_6	7.444
Diastereomers **1c**_6	4.174	Diastereomers **1f**_7	5.798
Diastereomers **1c**_7	2.028	Diastereomers **1f**_8	3.585
Diastereomers **1c**_8	1.796	Diastereomers **1f**_9	2.170
Diastereomers **1c**_9	1.639	Diastereomers **1f**_10	2.001
Diastereomers **1c**_10	1.332	Diastereomers **1f**_11	1.437
Diastereomers **1c**_11	0.859	Diastereomers **1f**_12	1.384
Diastereomers **1c**_12	0.736	Diastereomers **1f**_13	1.287
Diastereomers **1d**_1	52.368	Diastereomers **1f**_14	1.264
Diastereomers **1d**_2	13.017	Diastereomers **1f**_15	1.242
Diastereomers **1d**_3	12.848	Diastereomers **1f**_16	0.969
Diastereomers **1d**_4	8.856	Diastereomers **1f**_17	0.710
Diastereomers **1d**_5	3.632	Diastereomers **1f**_18	0.672
Diastereomers **1d**_6	3.439	Diastereomers **1f**_19	0.670
Diastereomers **1d**_7	2.437	Diastereomers **1f**_20	0.637
Diastereomers **1d**_8	2.017	Diastereomers **1f**_21	0.617
Diastereomers **1d**_9	1.386	Diastereomers **1f**_22	0.505
Diastereomers **1e**_1	47.032	Diastereomers **1f**_23	0.458
Diastereomers **1e**_2	32.018	Diastereomers **1f**_24	0.451
Diastereomers **1e**_3	8.848	Diastereomers **1f**_25	0.418
Diastereomers **1e**_4	7.223	Diastereomers **1f**_26	0.395
Diastereomers **1e**_5	2.479	Diastereomers **1f**_27	0.387
Diastereomers **1e**_6	2.400	Diastereomers **1f**_28	0.384
Diastereomers **1f**_1	20.504	Diastereomers **1f**_29	0.383

Table S2. Experimental and calculated chemical shift (δ_H) of diastereomers **1c** (4*R*, 5*S*, and 21*R*), **1d** (4*R*, 5*S*, and 21*S*), **1e** (4*S*, 5*R*, and 21*R*) and **1f** (4*S*, 5*R*, and 21*S*) of **1**.

Position	Exp. CS of **1**	Cal. CS of diastereomers **1c**	Cal. CS of diastereomers **1d**	Cal. CS of diastereomers **1e**	Cal. CS of diastereomers **1f**
C-1	126.500	125.100	125.320	125.930	126.420
C-2	135.750	135.060	136.230	136.310	134.210
C-3	130.420	125.570	128.510	128.520	127.000
C-4	123.830	124.660	123.590	122.550	123.530
C-5	128.670	128.510	126.440	125.530	126.780
C-6	128.570	127.000	129.060	133.460	129.830
C-7	29.130	28.290	31.660	29.400	28.420
C-8	34.060	37.100	37.970	38.310	37.530
C-9	68.090	70.070	74.470	72.430	71.100
C-10	140.790	144.390	147.770	142.860	145.200
C-11	123.830	123.710	121.110	124.940	123.960
C-12	126.540	126.230	127.970	126.190	126.980
C-13	17.720	26.280	26.150	24.510	25.440
C-14	126.430	126.810	130.290	128.300	125.960
C-15	32.250	34.180	38.140	35.730	38.020
C-16	75.840	78.890	80.280	76.870	82.160
C-17	33.410	36.650	38.640	36.000	39.750
C-18	173.980	176.630	175.360	175.730	176.410
C-19	46.990	49.590	46.480	45.630	47.130
C-20	148.620	150.880	153.320	149.070	151.360
C-21	113.940	119.660	121.330	119.910	121.900
C-22	41.140	46.880	48.160	46.840	47.760
C-23	17.350	25.060	23.750	17.950	24.940
C-24	31.980	36.050	34.930	33.950	34.040
C-25	86.900	91.350	91.930	84.940	83.600
C-26	36.710	42.790	39.240	45.580	44.380
C-27	139.220	139.820	139.580	140.390	140.720
C-28	127.330	129.620	127.780	129.380	128.980
C-29	16.580	21.000	23.010	20.560	20.790
C-30	134.440	132.750	133.280	132.190	131.890
C-31	115.490	117.909	117.700	118.640	118.790
C-32	105.870	115.530	112.720	109.360	105.630
C-33	75.470	77.890	77.640	80.120	74.430
C-34	78.030	81.480	81.420	80.360	82.040
C-35	71.870	73.310	74.280	74.110	73.350
C-36	75.140	81.360	81.500	84.540	81.130
C-37	64.880	67.710	67.960	67.930	66.720
H-1	6.440	5.800	5.420	5.410	5.480
H-2	6.000	5.540	5.420	5.260	5.370
H-3	6.160	5.830	5.500	5.360	5.530
H-4	6.440	5.880	5.760	5.670	5.840
H-5	5.710	5.130	5.160	5.200	5.090
H-6	1.940	1.730	1.830	1.220	1.140

H-7	2.300	1.950	1.850	1.690	1.510
H-8	1.730	1.470	0.830	1.380	1.290
H-9	1.800	1.500	1.610	1.530	1.420
H-10	4.670	4.200	4.110	4.140	4.230
H-11	6.230	5.580	5.410	5.580	5.570
H-12	1.800	0.950	0.970	0.950	0.780
H-13	1.800	1.530	1.550	1.440	1.390
H-14	1.800	1.820	1.730	1.950	1.860
H-15	6.200	5.640	5.820	5.590	5.640
H-16	5.250	4.930	5.410	4.940	4.860
H-17	2.390	1.840	1.800	1.350	1.720
H-18	2.590	1.960	1.980	2.740	2.210
H-19	4.800	4.030	3.880	4.170	3.670
H-20	3.050	2.390	2.190	2.490	2.450
H-21	3.440	2.490	2.510	2.530	2.600
H-22	5.040	4.740	4.810	4.530	4.940
H-23	5.040	4.670	4.610	4.460	4.740
H-24	0.970	0.720	0.540	0.670	0.660
H-25	0.970	0.520	0.520	0.290	0.560
H-26	0.970	0.730	0.720	1.160	0.700
H-27	5.630	4.980	5.400	5.300	5.180
H-28	2.500	1.670	1.920	1.560	1.890
H-29	2.730	2.500	1.980	1.790	2.060
H-30	3.690	3.060	3.170	3.160	3.390
H-31	2.370	1.750	1.680	3.310	1.540
H-32	1.700	1.110	1.370	1.000	1.040
H-33	1.810	1.240	1.420	1.660	1.410
H-34	2.100	1.490	1.610	1.620	1.620
H-35	2.100	1.540	1.800	1.780	2.090
H-36	1.770	0.940	0.900	1.110	1.110
H-37	1.770	1.250	1.340	1.290	1.120
H-38	1.770	1.530	1.550	1.590	1.600
H-39	5.850	5.280	5.260	5.340	5.340
H-40	6.580	6.020	5.980	5.920	5.850
H-41	4.960	4.480	4.500	4.420	4.450
H-42	5.060	4.500	4.510	4.540	4.710
H-43	3.180	2.780	2.780	2.950	3.030
H-44	3.300	3.130	3.180	3.210	3.150
H-45	3.260	3.110	2.910	2.960	3.250
H-46	4.280	3.640	3.560	4.020	3.810
H-47	3.420	2.920	2.820	3.210	2.840
H-48	4.220	3.450	3.250	3.460	3.420
H-49	4.400	3.760	3.630	3.810	3.670

Figure S17. Statistical comparison of the calculated and experimental chemical shifts by the tool of DP4 calculation. (Isomer 1: **1c** (4*R*, 5*S*, and 21*R*), Isomer 2: **1d** (4*R*, 5*S*, and 21*S*), Isomer 3: **1e** (4*S*, 5*R*, and 21*R*), Isomer 4: **1f** (4*S*, 5*R*, and 21*S*))

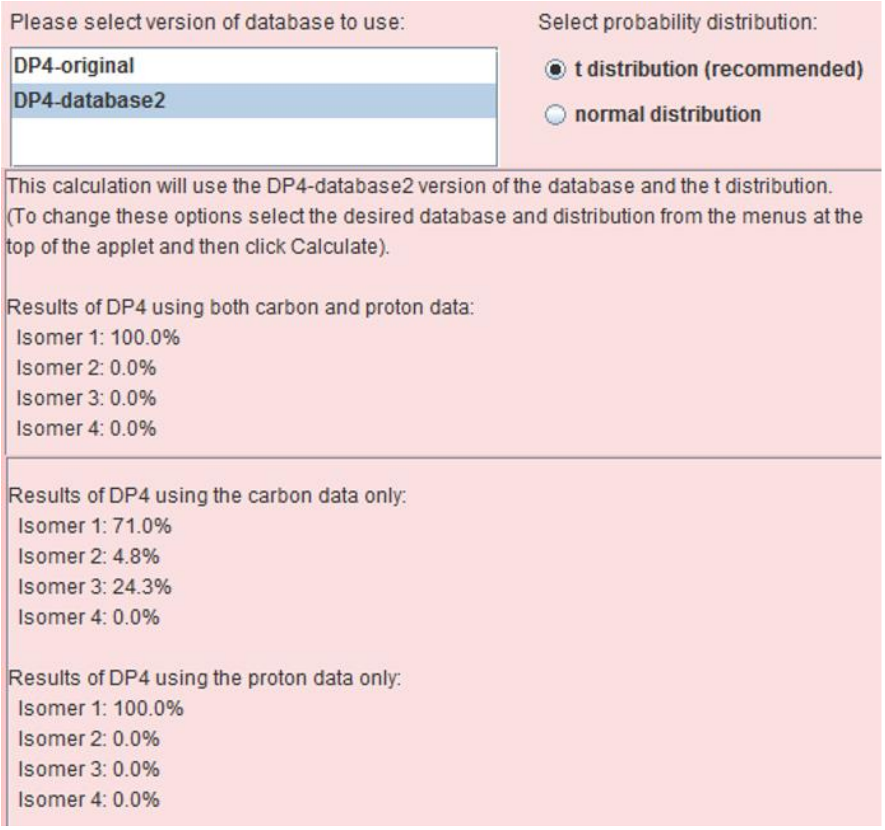

Figure S18. ^{1}H NMR spectrum of **2** at 800 MHz in CD_3OD.

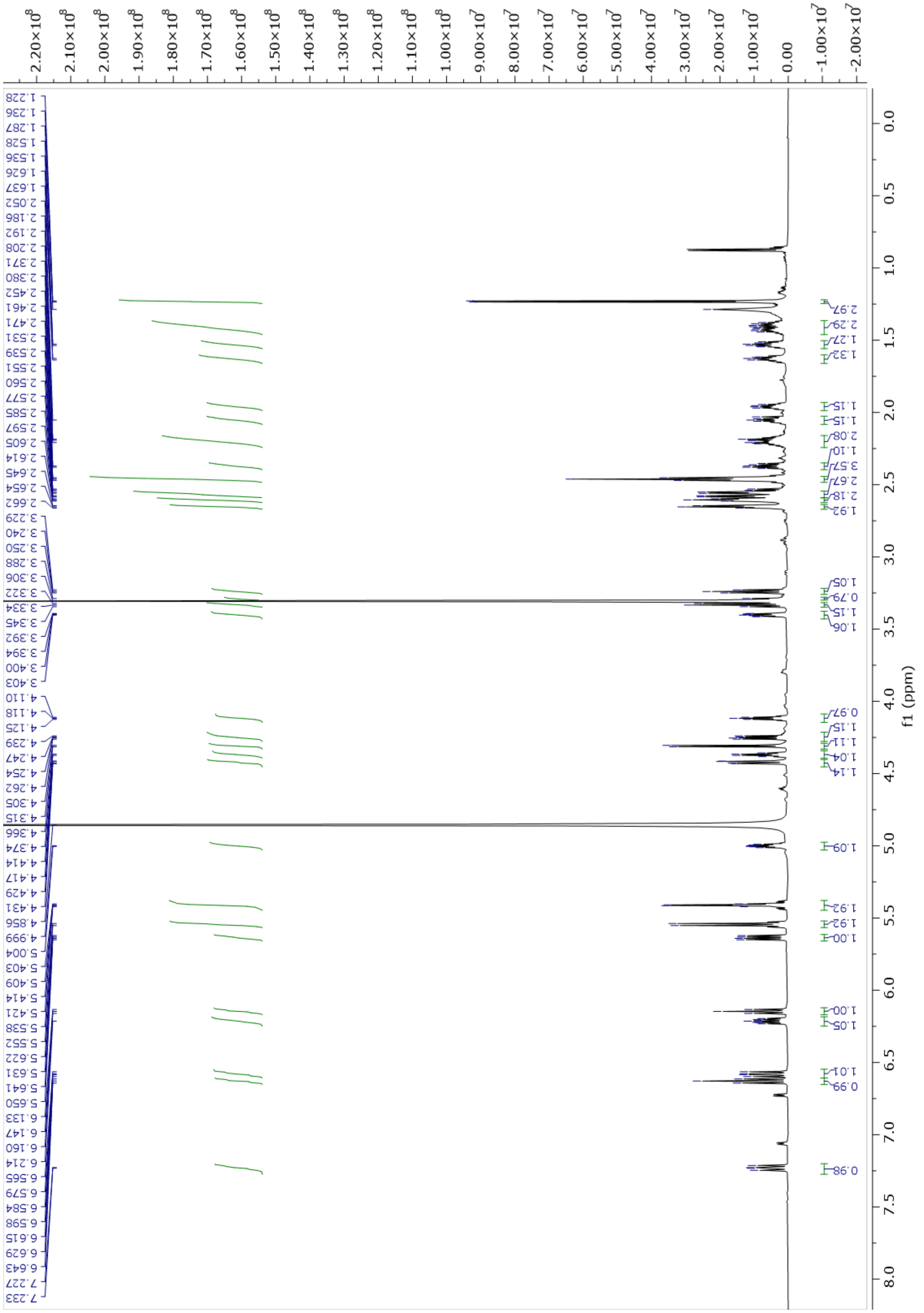

Figure S19. ^{13}C NMR spectrum of **2** at 800 MHz in CD_3OD.

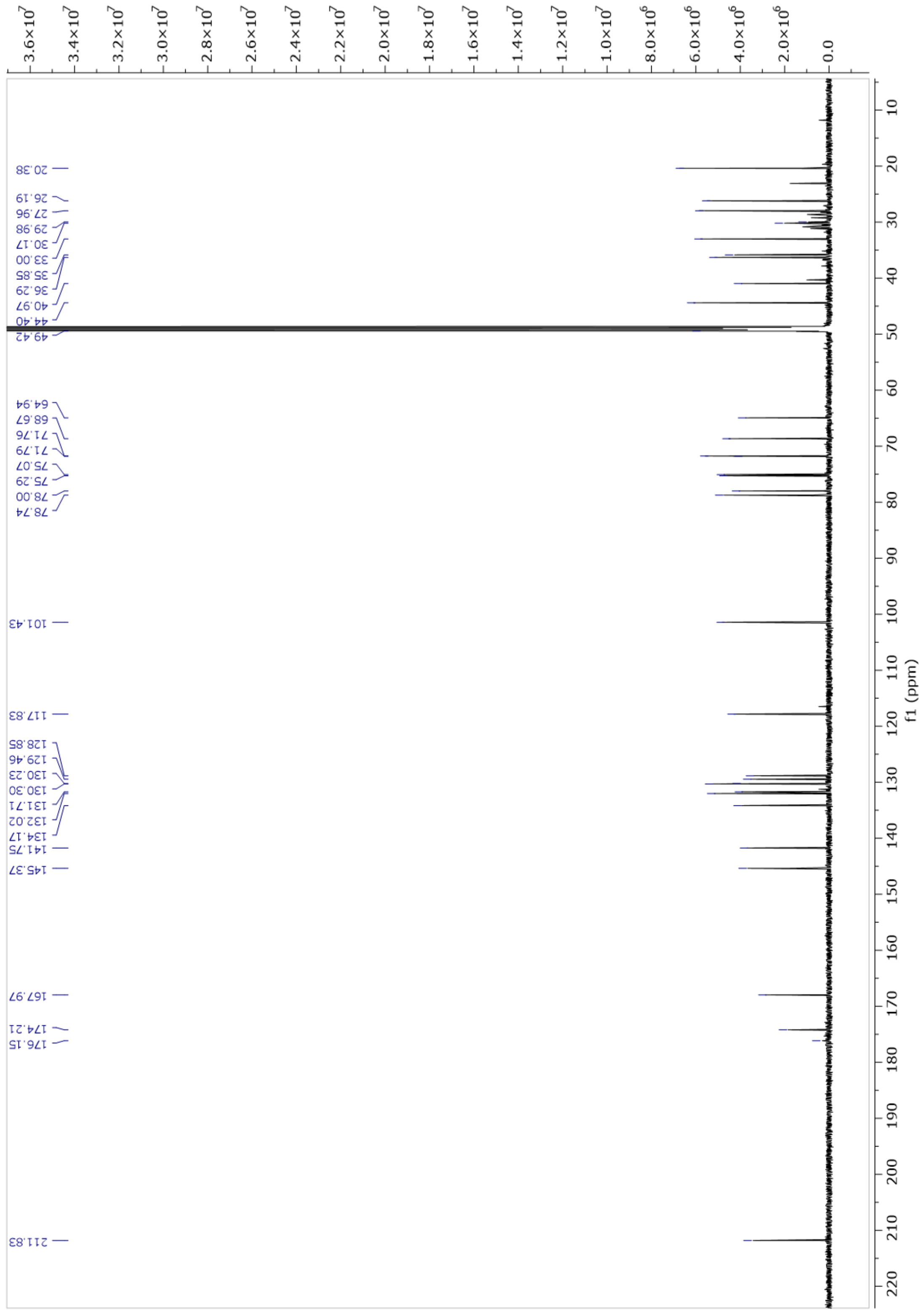

Figure S20. COSY spectrum of **2** at 800 MHz in CD_3OD.

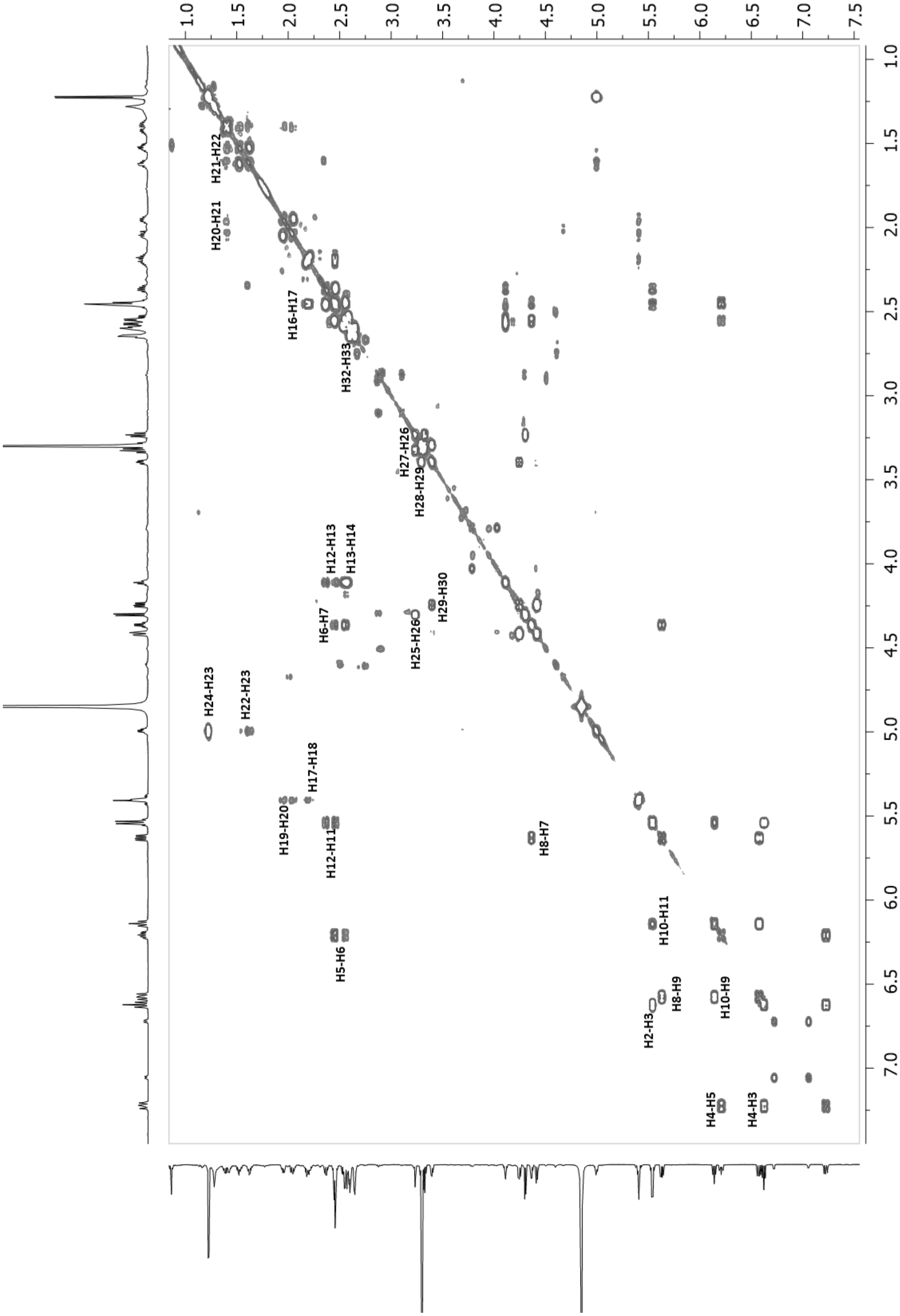

Figure S21. HSQC spectrum of **2** at 800 MHz in CD_3OD.

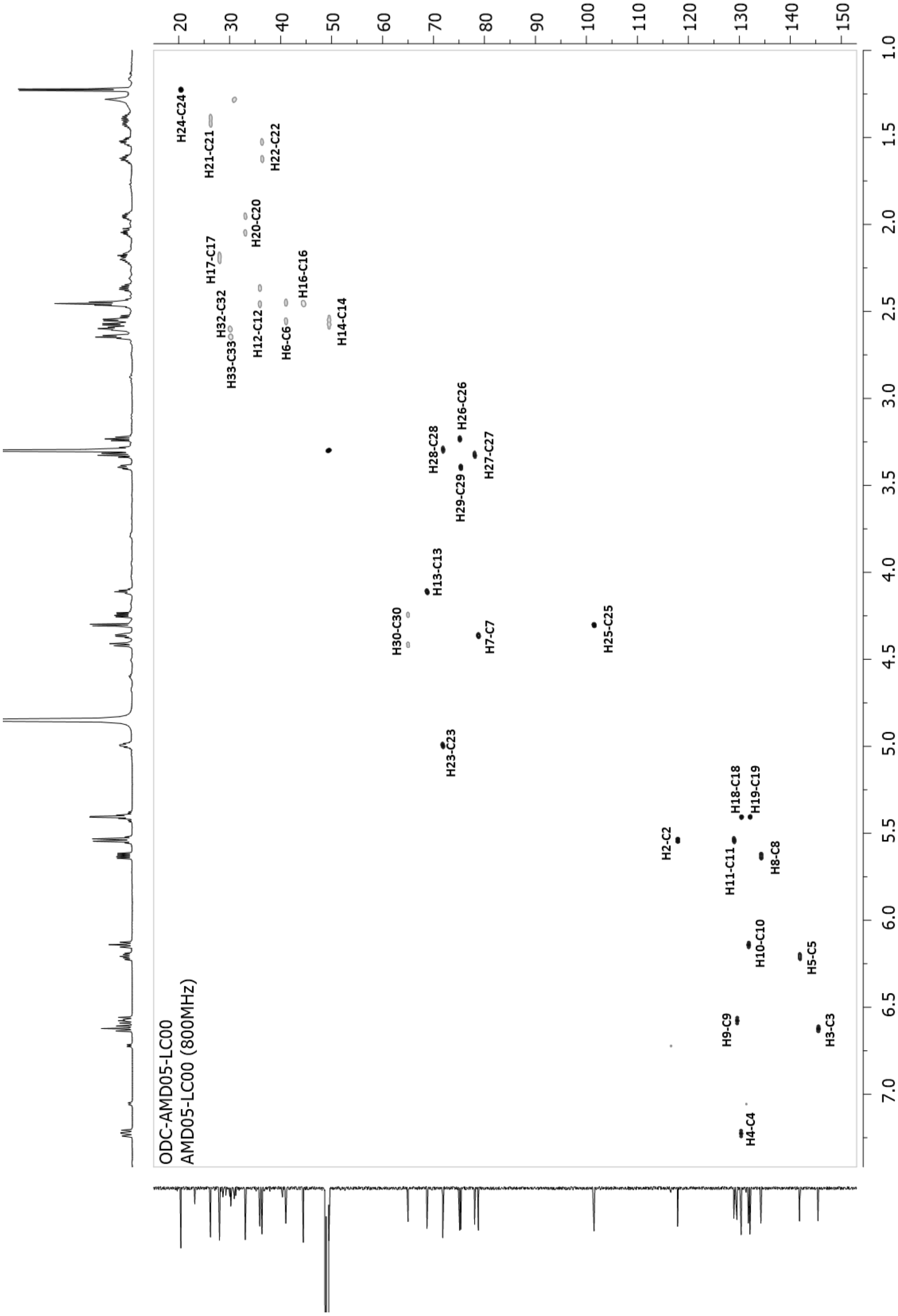

Figure S22. HMBC spectrum of **2** at 800 MHz in CD_3OD.

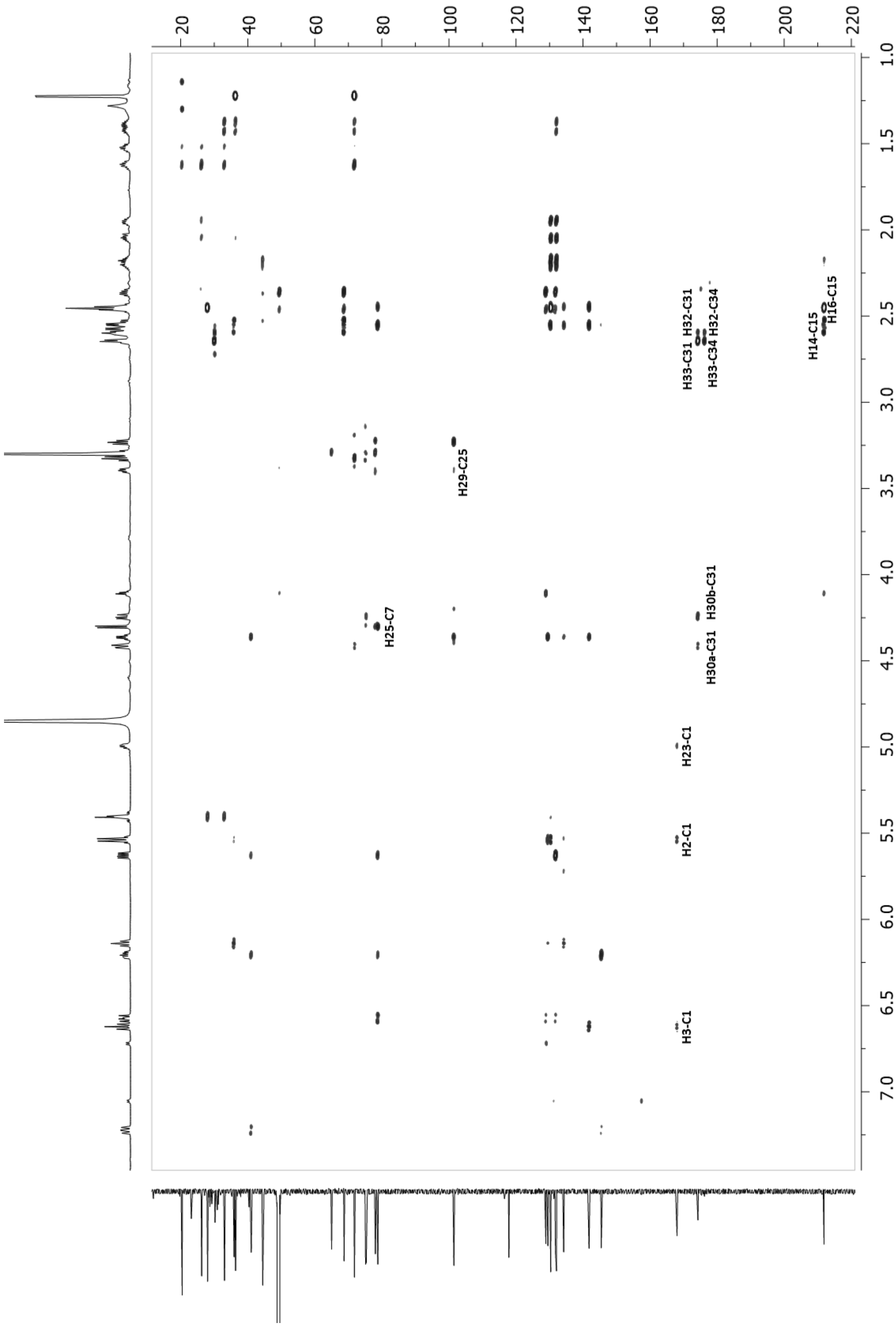

Figure S23. ROESY spectrum of **2** at 800 MHz in CD_3OD.

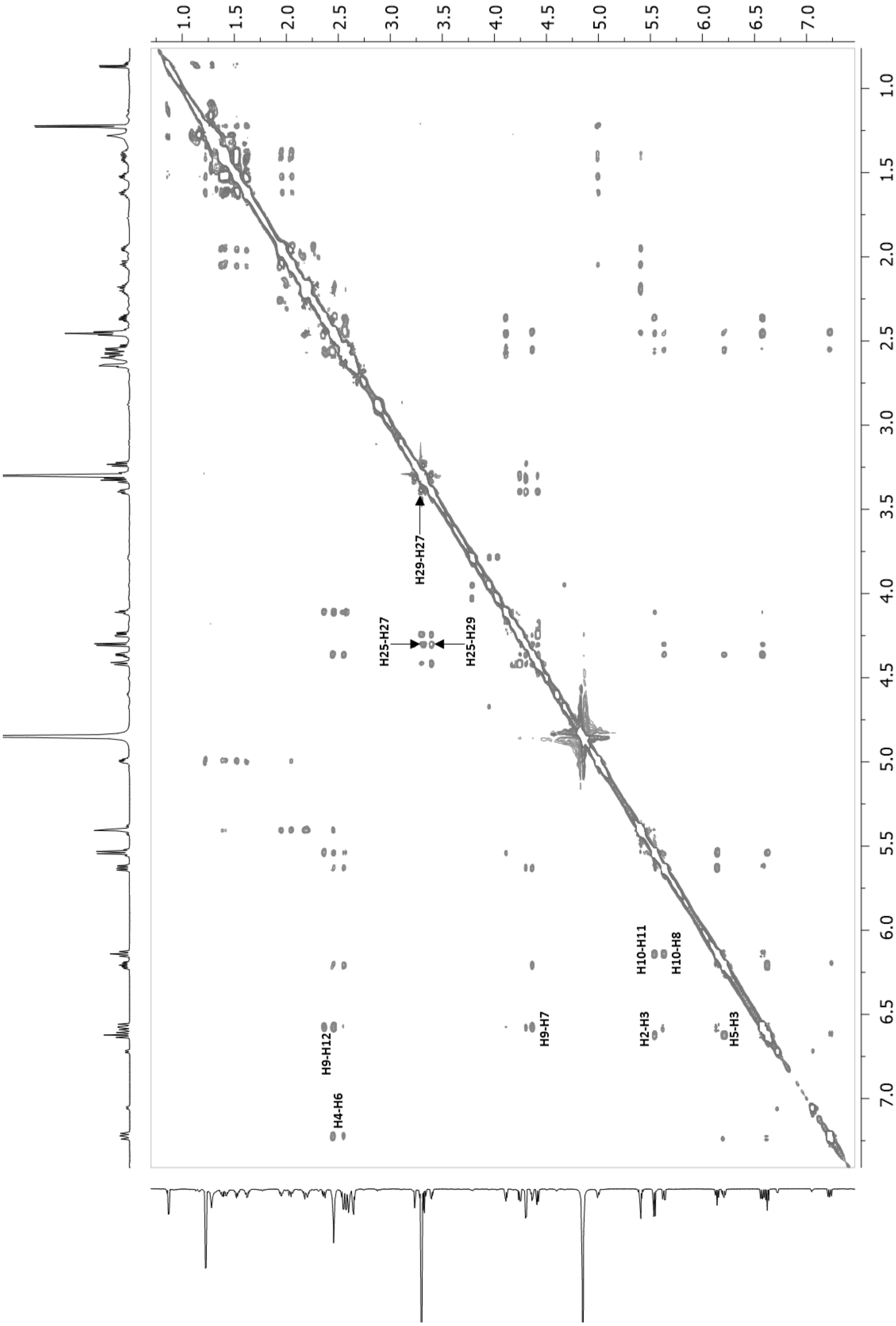

Figure S24. UV spectrum of **2**.

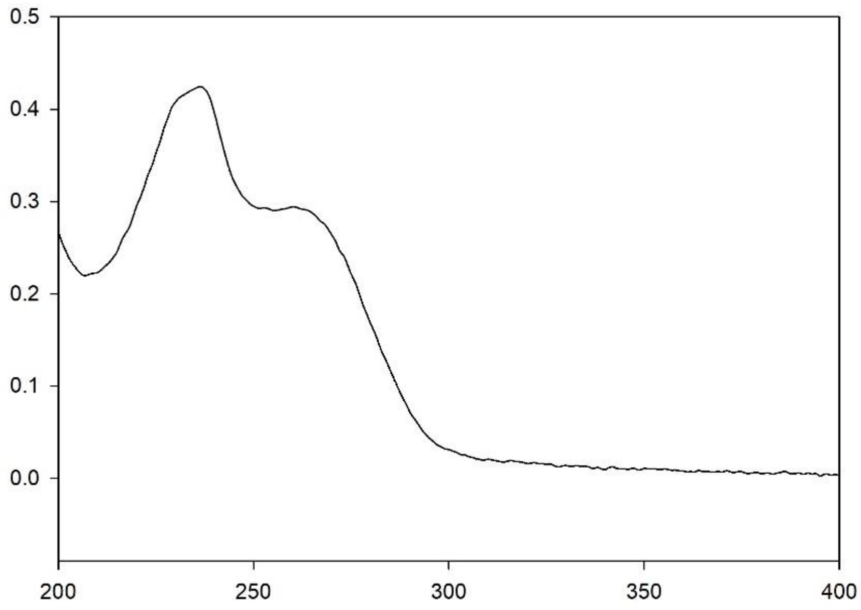

Figure S25. CD spectrum of **2**.

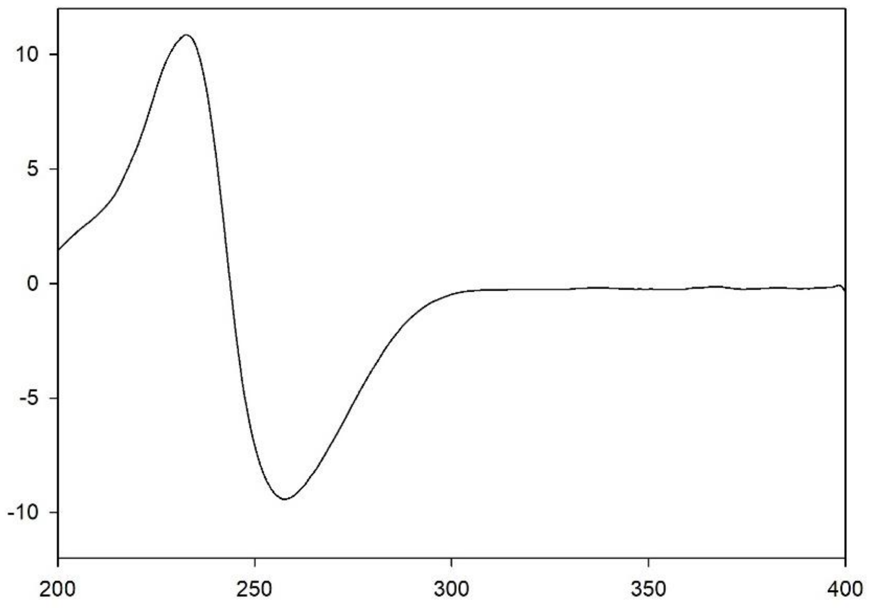

Figure S26. IR spectrum of **2**.

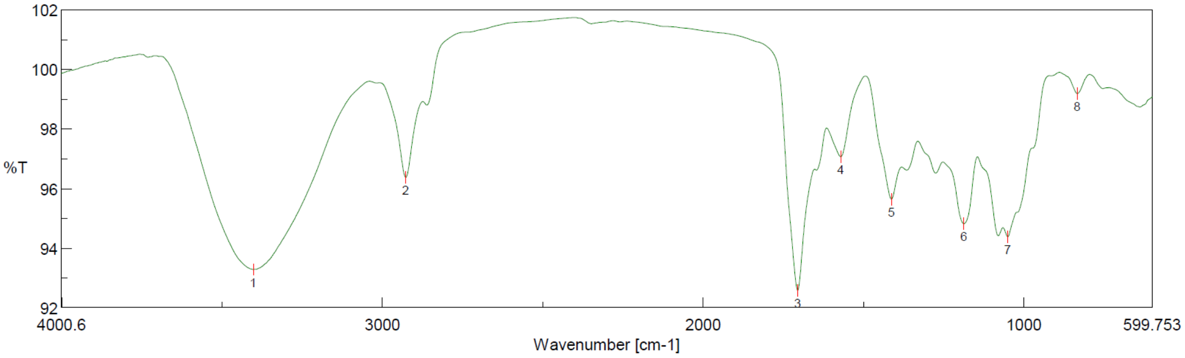

[Data Information]

Creation Date	2021-06-03 오후 3:30
Data array type	Linear data array
Horizontal	Wavenumber [cm-1]
Vertical	%T
Start	599.753 cm-1
End	4000.6 cm-1
Data pitch	0.964233 cm-1
Data points	3528

[Result of Peak Picking]

No.	Position	Intensity	No.	Position	Intensity	No.	Position	Intensity	No.	Position	Intensity
1	3402.78	93.2831	2	2927.41	96.3741	3	1704.76	92.5815	4	1570.74	97.0665
5	1412.6	95.643	6	1186.97	94.8193	7	1050.05	94.3809	8	833.098	99.1872

Figure S27. HR-ESI-MS spectrum of **2**.

Spectrum from AMD05_MW664.wiff (sample 1) - AMD05_MW664, Experiment 1, +TOF MS (100 - 2000) from 0.435 min

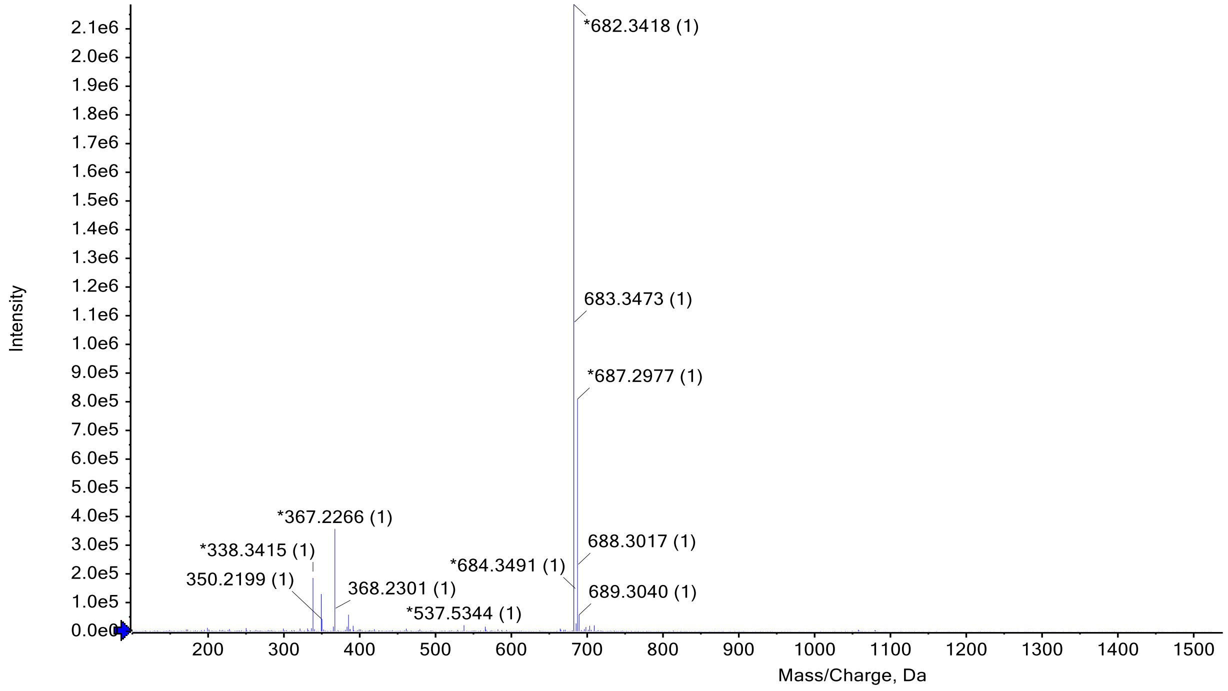

Figure S28. LC/MS analysis of β-glucopyranose reaction product from **2** coinjecting with each authentic β-L-glucose reaction product and authentic β-D-glucose reaction product.

β-glucopyranose reaction product of **2** + authentic β-L-glucose reaction product coinjection:

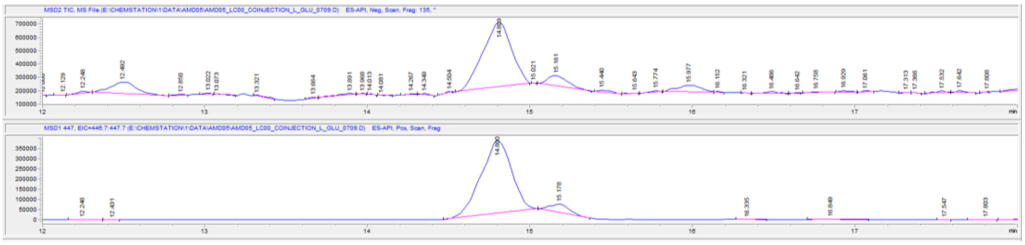

β-glucopyranose reaction product of **2** + authentic β-D-glucose reaction product coinjection:

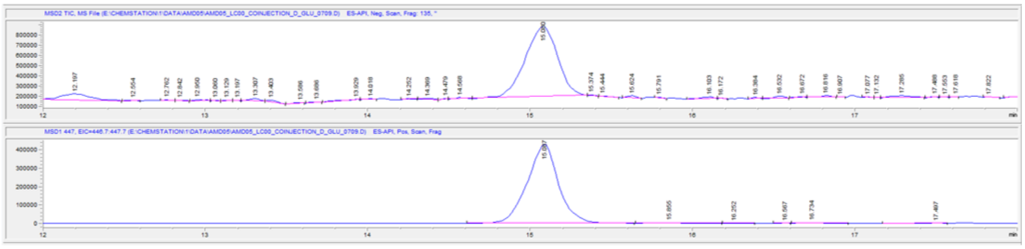

The sugar was revealed as β-D-glucose.
